# Selective and direct hydrogen generation from mixed plastic waste via alkaline thermal treatment with inherent carbon storage

**DOI:** 10.1073/pnas.2537552123

**Published:** 2026-07-06

**Authors:** Jieun Park, Hyunah Kim, Hyerin Seo, Jiwon Lee, Hyung-Kyu Lim, Wonho Jung, Ah-Hyung Alissa Park, Woo-Jae Kim

**Affiliations:** ^a^ Department of Chemical Engineering and Materials Science, Graduate Program in System Health Science and Engineering, Ewha Womans University, Seoul 03760, Republic of Korea; ^b^ Department of Materials Science and Engineering, Korea Aerospace University, Gyeonggi-do 10540, Republic of Korea; ^c^ Division of Chemical Engineering and Bioengineering, Kangwon National University, Chuncheon, Gangwon-do 24341, Republic of Korea; ^d^ C1 Gas Refinery Research and Development Center, Sogang University, Seoul 04107, Republic of Korea; ^e^ Department of Chemical and Biomolecular Engineering, University of California, Los Angeles, Samueli School of Engineering, Los Angeles, CA 90095; ^f^ Institute for Multiscale Matter and Systems, Ewha Womans University, Seoul 03760, Republic of Korea

**Keywords:** plastic recycling, alkaline thermal treatment, hydrogen production, catalysts, carbon capture

## Abstract

The global accumulation of plastic waste has spurred extensive research into chemical recycling methods to mitigate environmental issues and convert waste into valuable resources. A major challenge in plastic recycling is the requirement for presorting, which complicates processing and increases costs. Here, we demonstrate alkaline thermal treatment (ATT) as a highly efficient strategy for directly converting mixed plastic waste, including polyethylene terephthalate (PET), polyethylene (PE), and polypropylene (PP), into clean hydrogen energy at low temperatures and atmospheric pressure. Unlike conventional gasification, NaOH-assisted ATT enables plastic decomposition at significantly lower temperatures while producing high-purity hydrogen and minimizing carbon emissions. A key advancement in this work is the oxidation pretreatment of PP and PE, which enhances their reactivity in ATT and allows efficient hydrogen generation even from typically resistant polyolefins. Through systematic optimization of the NaOH-to-plastic ratio and thermal oxidation conditions, hydrogen yields of 43.7, 51.9, and 30.2 mmol/g_plastic_ were achieved for PET, PE, and PP, respectively. Furthermore, ATT efficiently converts both individual and mixed plastic waste without requiring extensive separation, demonstrating its commercial potential and scalability with real-world waste compositions. Overall, this study establishes ATT as a promising and sustainable solution for plastic waste management and clean energy production, providing an economically viable low-carbon pathway for hydrogen generation.

## Significance

Plastic waste is difficult to recycle because mixed plastics typically require sorting and high-temperature gasification that emits CO_2_. We demonstrate an alkaline thermal treatment (ATT) that converts common mixed plastics such as polyethylene terephthalate (PET), polyethylene (PE), and polypropylene (PP) into high-purity hydrogen at substantially lower temperatures while suppressing carbon-containing byproducts. A simple thermal-oxidation pretreatment activates PE and PP by introducing oxygen functional groups, making them reactive in ATT. First-principles calculations reveal that the introduced oxygen functional groups lower reaction barriers in alkaline media, explaining the activation of oxidized PE and PP. By combining lower-temperature operation, carbon capture capability, and advantages of mixed plastics, the ATT offers a practical pathway to upcycle plastic waste into clean hydrogen fuel.

Plastics have become indispensable in modern society owing to their exceptional properties, which include durability, versatility, and lightweight structure. These attributes make them essential in various industries, including packaging, construction, healthcare, and consumer goods. Consequently, global plastic production has surged, reaching approximately 400 million tons in 2022 (1–3). However, their inherent resistance to degradation poses severe environmental challenges. Plastics have persisted in the natural environment for centuries, accumulating at alarming rates and substantially contributing to global pollution (1, 4–8). The problem is further worsened by the complexity of mixed plastic waste, where different polymer types are discarded together, making conventional recycling methods inefficient (9–12). Addressing this issue requires innovative strategies capable of directly processing mixed plastic waste without presorting.

Despite the increasing emphasis on recycling, only 9% of plastic waste is currently recycled, whereas 79% accumulates in landfills and 12% undergoes incineration (13, 14). Traditional waste management practices not only fail to adequately address the plastic waste crisis but also contribute to secondary environmental pollution, including the release of microplastics, fine particulates, SO_x_, NO_x_, dioxins, and CO_2_. Consequently, the development of sustainable and efficient plastic waste recycling technologies is an urgent priority (9, 15–19).

Thermochemical recycling, particularly pyrolysis and gasification, has gained significant attention as a method for converting plastic waste into valuable energy resources (9, 10, 20–24). Pyrolysis decomposes plastics in an oxygen-free environment, yielding liquid oil, gaseous products, and char. Although it is considered an environmentally favorable process because of its lower carbon emissions, its application is limited to certain plastic types and requires extensive separation and refining to achieve high-quality outputs. In contrast, gasification partially oxidizes plastics at high temperatures to produce syngas, a mixture of hydrogen, carbon monoxide, and hydrocarbons. In the gasification process, mixed plastic wastes are processed without extensive sorting, therefore, it is considered a more economically viable approach. Consequently, research has increasingly focused on continuous pyrolysis-gasification systems. For instance, Jinhu Li et al. investigated the copyrolysis and cogasification of polypropylene (PP) and polystyrene (PS), while Chunfei Wu et al. demonstrated hydrogen production through steam pyrolysis of PP at 500 °C, followed by continuous gasification at 800 °C using a Ni-Mg-Al catalyst (25, 26). These studies highlighted the role of hydrocarbon steam reforming and water-gas shift reactions in enhancing hydrogen production, alongside the formation of CO and CO_2_, facilitated by metal catalysts (27–29). However, conventional gasification remains highly energy-intensive, typically requiring high pressures (MPa) and extreme temperatures (800 to 1,000 °C) to break down plastics, resulting in substantial CO_2_ emissions. This highlights the need for alternative energy-efficient technologies capable of effectively converting plastic waste into hydrogen while minimizing environmental impact.

To address these challenges, we propose an innovative approach using alkaline thermal treatment (ATT) for presorting-free plastic waste recycling—an emerging alternative initially developed for biomass conversion, particularly for hydrogen production (30–36). By employing NaOH as an alkaline reagent, ATT significantly lowers the reaction temperature, enhances hydrogen yield, and minimizes CO_2_ emissions compared with conventional thermochemical methods. In our previous study, ATT successfully converted seaweed biomass into hydrogen, achieving a maximum yield of 69.69 mmol H_2_ per gram of seaweed (31). During the reaction, carbon was captured as Na_2_CO_3_, and NaOH was regenerated via a Ca(OH)_2_-assisted recovery process, further enhancing the sustainability of this method.

Building on this concept, we applied ATT to widely used plastic waste streams, including polyethylene terephthalate (PET), polyethylene (PE), and polypropylene (PP). Our findings demonstrate that in the presence of NaOH, ATT operates at significantly lower temperatures while achieving higher hydrogen production rates than traditional gasification. This enhancement is attributed to the catalytic role of NaOH, which facilitates polymer degradation under milder conditions. Furthermore, by comparing the hydrogen production efficiencies of PET (which contains carbon, hydrogen, and oxygen functional groups) with those of PE and PP (which contain only carbon and hydrogen), we elucidate the crucial role of oxygen functional groups in improving reactivity during ATT. Based on these insights, we optimized the NaOH-to-plastic ratio and introduced a thermal oxidation pretreatment for PE and PP, which substantially improved the hydrogen yield. Notably, we also found that mixed plastic waste composed of PET, PE, and PP can be efficiently converted into hydrogen via ATT without requiring extensive presorting, thus addressing a major limitation of conventional recycling processes. These results highlight the direct applicability of ATT in treating real-world plastic waste streams while achieving high hydrogen yields from mixed plastics.

Overall, this study establishes ATT as a scalable and sustainable approach to the management of plastic waste and production of clean energy. By enabling high-purity hydrogen generation while minimizing carbon emissions, ATT offers a practical and effective solution to the global plastic waste crisis, bridging the gap between recycling and hydrogen-based energy sustainability.

## Results and Discussion

### H_2_ Production from ATT Using PET Plastic.

**Fig. 1.**
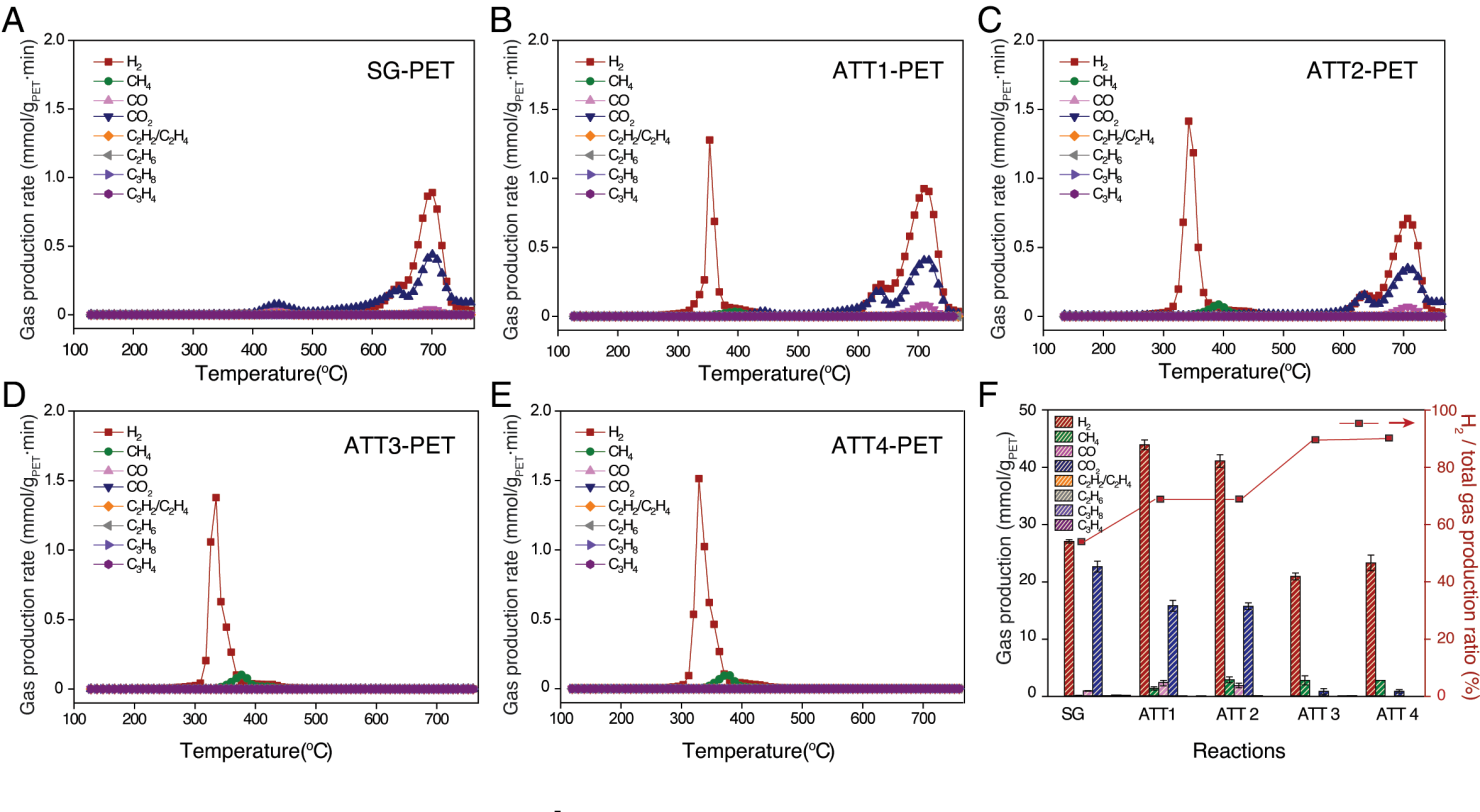
Gas production rates during the decomposition of PET under different reaction conditions. Comparison of hydrogen production between SG-PET and ATT-PET with various NaOH-to-PET mass ratios. Real-time gas production profiles are shown for (*A*) SG-PET, (*B*) ATT with a NaOH-to-PET ratio of 1 (ATT1-PET), (*C*) ATT with a NaOH-to-PET ratio of 2 (ATT2-PET), (*D*) ATT with a NaOH-to-PET ratio of 3 (ATT3-PET), and (*E*) ATT with a NaOH-to-PET ratio of 4 (ATT4-PET). The detected gaseous products include H_2_, CH_4_, CO, CO_2_, C_2_H_4_/C_2_H_6_, C_3_H_6_, and C_4_H_10_. (*F*) Overall gas production under each reaction condition, with hydrogen production efficiency represented as the H_2_-to-total gas production ratio. Error bars represent the SD of three independent samples.

To evaluate the hydrogen production potential of plastic waste recycling, we first investigated SG using PET as a representative feedstock due to its significant contribution to global plastic waste (37). As shown in Fig. 1*A*, hydrogen production during steam gasification of PET (SG-PET) occurred primarily near 700 °C, yielding 26.9 mmol/g_PET_. Building on this, we employed ATT as an alternative strategy, leveraging its proven efficiency in biomass conversion and hydrogen production. With an initial NaOH-to-PET mass ratio of 1:1, ATT1 significantly outperformed SG by initiating hydrogen production at substantially lower temperatures (300 to 400 °C), achieving an average hydrogen yield of 43.7 mmol/g_PET_ as seen in Fig. 1*B*. This result demonstrates the superior efficiency of ATT over traditional SG for hydrogen production from PET plastic waste, highlighting the importance of ATT in facilitating this process. A secondary hydrogen production peak near 700 °C, indicates that a portion of PET carbon remained unconverted at lower temperatures and underwent additional thermal decomposition at elevated temperatures. This indicates that although NaOH catalyzes PET depolymerization and hydrolysis, the initial loading may be insufficient for complete conversion into Na_2_CO_3_ (27–34). To optimize the process, we performed additional ATT reactions by increasing the NaOH-to-PET mass ratios of 2:1, 3:1, and 4:1 for ATT2-PET, ATT3-PET, and ATT4-PET, respectively (Fig. 1 *C*–*E*). ATT2-PET demonstrated an increase in hydrogen production near 300 °C while suppressing high-temperature gas production. Notably, the production of carbon-based byproducts such as CH_4_, CO, and CO_2_ at lower temperatures was significantly reduced, indicating a selective hydrogen production pathway. ATT2-PET achieved a hydrogen yield of 40.9 mmol/g_PET_, reinforcing role of NaOH in shifting the reaction mechanism toward low-temperature hydrogen production. This result agrees with previous studies demonstrating that NaOH promotes PET depolymerization and enhances hydrogen production (38). Increasing the NaOH-to-PET ratio (ATT3-PET and ATT4-PET) effectively eliminated high-temperature gas emissions, confirming that higher NaOH concentrations facilitate PET conversion at ~300 °C. Although the hydrogen yields of ATT3-PET and ATT4-PET (20.8 and 23.1 mmol/g_PET_, respectively) did not significantly increase with additional NaOH, Fig. 1*F* shows that both the hydrogen-to-total gas ratio and the hydrogen-to-carbon-based product ratio increased with increasing NaOH concentrations, indicating a more efficient hydrogen production pathway primarily by reducing carbon-based byproducts such as CO_2_. These results underscore the critical role of NaOH in facilitating low-temperature hydrogen production from PET waste, highlighting ATT as a promising strategy for sustainable plastic waste recycling.

Interestingly, ATT1-PET produced the highest hydrogen yield, even compared to reactions with higher NaOH loadings. In a higher NaOH-to-PET ratio, the overall hydrogen yield decreased because high-temperature hydrogen generation was suppressed. This suggests that, at elevated NaOH loadings, a portion of hydrogen remains incorporated within intermediate products, such as light hydrocarbons, tar, or char, rather than being released as gaseous hydrogen, consistent with the observations of Li et al. (39). This behavior indicates a shift in the PET decomposition pathway under ATT conditions, from high-temperature thermal decomposition to low-temperature hydrolysis and reforming, governed by the presence of NaOH. Consequently, a portion of hydrogen becomes trapped within solid residues (char) or liquid (ethylene glycol, functional groups in carboxylic acids, alcohols), thereby reducing the amount of hydrogen released as gas and ultimately lowering the overall yield. To further elucidate these reaction pathways involved in alkaline thermal treatment of PET (ATT-PET), we conducted a detailed analysis of the gas products. As shown in Fig. 1, in addition to hydrogen production, CO_2_, CH_4,_ and CO were detected under various reaction conditions. In SG-PET, the formation of H_2_, CO, CO_2_, and CH_4_ results from the thermal decomposition of aromatic rings and ester bonds at elevated temperatures, consistent with previous reports (39, 40). Additionally, secondary reactions such as the steam methane reforming further contribute to CO_2_ generation, resulting in substantial carbon-containing byproducts. In contrast, ATT-PET follows a distinct reaction pathway. Instead of undergoing direct thermal decomposition, PET reacts with NaOH, to form sodium carbonate (Na_2_CO_3_), which stabilizes carbonates and suppresses CO and CO_2_ production (41). This process enables high-purity hydrogen production at significantly lower temperatures (300 to 400 °C), making ATT more selective and efficient than SG. Additionally, CH_4_ was detected as a minor byproduct in ATT-PET, particularly at lower temperatures (Fig. 1). This phenomenon is attributed to the reduction of carbon intermediates in the presence of abundant hydrogen, which is consistent with previous reports (42). These findings suggest that low-temperature hydrogenation reactions are active in ATT, further distinguishing its mechanism from that of high-temperature SG.

Overall, ATT enhances hydrogen production efficiency while suppressing undesirable carbon-containing byproducts, offering a promising pathway for clean hydrogen production from PET waste.

### Possible Reaction Mechanism of ATT-PET for Hydrogen Production.

The overall reaction of ATT-PET is described as

[1] $$\begin{array}{l} \left[{\text{C}}_{10}{\text{H}}_{8}{\text{O}}_{4}\right]\;+\;20\;\text{NaOH}\\ \;\;\;\;\;\;\;\;\;\;\;+\;6\;{\text{H}}_{2}\text{O}\to 20\;{\text{H}}_{2}↑+\;10\;{\text{Na}}_{2}{\text{CO}}_{3}. \end{array}$$

Based on the overall stoichiometry of the reaction in Eq. **1**, the actual origin of hydrogen may involve contributions from both PET and H_2_O. If only the hydrogen atoms initially contained in PET are considered, the maximum theoretical hydrogen yield is 0.0208 mol/g_PET_ whereas the overall stoichiometric yield, including the participation of H_2_O is 0.104 mol/g_PET_. In addition, because hydrogen-containing gaseous products such as CH_4_ are also formed under some conditions, the hydrogen balance cannot be interpreted based on H_2_ alone. Therefore, PET is considered a major hydrogen source, while the contribution of H_2_O cannot be excluded.

The reaction begins with PET depolymerization, where NaOH cleaves the ester bonds, generating terephthalate-containing salts together with hydroxyethyl-terminated fragments and other PET-derived oxygenated intermediates (*SI Appendix*, Fig. S1). The alkaline environment significantly lowers the activation energy required for depolymerization. Literature reports show that alkaline hydrolysis of PET occurs at 100 to 200 °C with an activation energy of ~26 kJ/mol, whereas neutral hydrolysis requires 300 to 400 °C with activation energies of ~74 kJ/mol (43, 44). This approximately threefold reduction in activation energy allows the reaction to proceed efficiently at substantially lower temperatures (41, 42).

Following the initial ester cleavage, PET likely passes through a distribution of oxygenated intermediates under thermal and alkaline conditions, including partially depolymerized oligomeric fragments and transient alcohol- and/or carbonyl-containing species. These PET-derived oxygenated intermediates can undergo further base-promoted decomposition and reforming reactions under ATT conditions, contributing to H_2_ evolution. Simultaneously, the generated CO_2_ reacts with NaOH to form Na_2_CO_3_, effectively capturing CO_2_ and preventing its release.

This integrated mechanism ensures high-purity hydrogen production while minimizing carbon-based byproducts. The ATT-PET process not only maximizes hydrogen yield but also incorporates an effective carbon capture mechanism that converts CO_2_ into stable carbonates. This dual functionality positions ATT-PET as an environmentally sustainable and energy-efficient method for converting plastic waste into valuable hydrogen fuel while mitigating carbon emissions.

### H_2_ Production from ATT Using PE and PP Plastics.

**Fig. 2.**
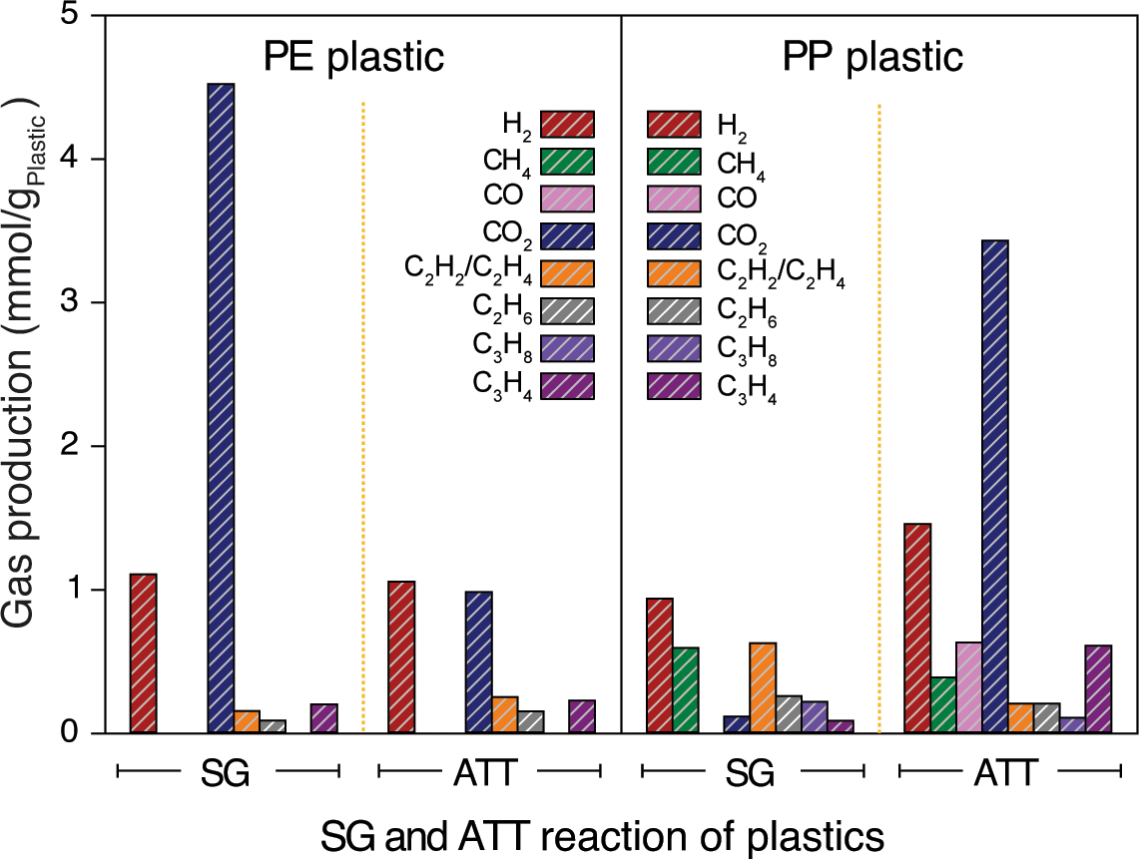
Gas production of PE and PP during SG and ATT reactions. Comparison of gas species generated from PE and PP under SG and ATT reactions. For ATT, a NaOH-to-plastic ratio of 4 was applied. The gas products of H_2_, CH_4_, CO_2_, CO, C_2_H_2_/C_2_H_4_, C_2_H_6_, C_3_H_6_, and C_3_H_4_ are shown.

Based on the PET results, the scope of plastic waste was expanded to include the next most widely used polymers, namely PE and PP. To investigate their decomposition and gas production behaviors, we first conducted SG to compare the properties of PET, PE, and PP. As shown in *SI Appendix*, Fig. S2, the gas production varied significantly among these plastics, revealing two distinct patterns based on the major components. PET exhibited the highest total gas yield (49.7 mmol/g_PET_), whereas PE and PP generated considerably lower amounts of 5.7 mmol/g_PE_ and 2.6 mmol/g_PP_, respectively. A similar trend was observed in the ATT process (Fig. 2), where PE and PP produced significantly lower total gas yields compared to PET (45). These differences can be attributed to variations in the polymer structure and functional group composition. PET contains oxygen functional groups in its repeating unit (C_10_H_8_O_4_)_n_, which facilitate hydrolysis under alkaline conditions, leading to the release of CO and CO_2_. In contrast, PE [(C_2_H_4_)_n_] and PP [(C_3_H_6_)_n_] consist exclusively of carbon-hydrogen bonds, and they exhibit high thermal degradation resistance. This thermal stability is further supported by their high decomposition energies. The decomposition energies range from 238 to 247 kJ/mol for high-density polyethylene (HDPE), 215 to 221 kJ/mol for low-density polyethylene (LDPE), and 179 to 188 kJ/mol for PP (46). Due to their chemical inertness and lack of oxygen-containing functional groups, PE and PP show low reactivity with NaOH, inhibiting hydrolysis and decomposition in the ATT process, resulting in minimal gas production under alkaline thermal conditions. To overcome this limitation and enhance the reactivity of PE and PP, a thermal oxidation pretreatment was applied before the ATT process. This approach introduces oxygen-containing functional groups into the polymer backbone, to promote alkaline hydrolysis and improve gas production efficiency.

### Thermal Oxidation of PE and PP Plastics.

To enhance the reactivity of PE and PP in the ATT process, thermal oxidation was conducted at temperatures ranging from 150 to 350 °C, exceeding their melting points. This process induced significant compositional modifications and was analyzed using FT-IR and elemental analysis (EA).

The FT-IR spectra shown in *SI Appendix*, Fig. S3 confirm the incorporation of oxygen-containing functional groups in thermally oxidized PE and PP (47–49). A distinct absorption peak at 1,718 cm^−1^ indicates carbonyl (C = O) groups, which serve as primary initiation sites for ATT. Additionally, broad bands in 3,300 to 3,600 cm^−1^ and the peak at 1,167 cm^−1^ correspond to O–H and C–O stretching vibrations, respectively, which are indicative of hydroxyl and ether functionalities. The introduction of these oxygen groups enhances the polymer reactivity during ATT. Notably, the emergence of a peak at 1,595 cm^−1^ suggests C = C bond formation, which, while not directly linked to oxidation, may further promote ATT by providing additional reaction sites. The thermal oxidation process of PE and PP primarily involves chain scission mediated by peroxy radicals, resulting in the formation of various oxygen-containing functional groups. Among these, ketones represent a predominant product (50, 51). This is consistent with our FT-IR observations showing the strong carbonyl (C = O) absorption at 1,718 cm^−1^. The formation of these ketone groups is particularly significant as they serve as active sites for subsequent reactions during the ATT process. Under strongly alkaline conditions, these carbonyl-containing oxidized segments may undergo competing base-mediated transformations, including aldol-type condensation and dehydration as well as retro-aldol-type fragmentation and related chain-scission reactions (52–54). This transformation creates structurally diverse intermediate species with varying thermal stability, which directly influences the hydrogen evolution profile observed during ATT. Collectively, FT-IR analysis demonstrates that thermal oxidation effectively modifies the chemical structure of PE and PP, increasing their susceptibility to subsequent ATT reactions. The EA further quantifies these compositional changes. As shown in *SI Appendix*, Figs. S4 and S5, a progressive increase in oxygen content was observed with increasing oxidation temperature for both PE and PP, corroborating the FT-IR results. This oxygen incorporation aligns with the functional group modifications detected via FT-IR, reinforcing the role of thermal oxidation in enhancing polymer reactivity.

Overall, the combined FT-IR and EA results demonstrate that thermal oxidation significantly alters the chemical structures of PE and PP by introducing oxygen-containing functional groups that facilitate polymer decomposition and hydrogen production in ATT. These findings underscore the critical role of preoxidation treatment in optimizing hydrogen production from hydrocarbon-based plastic waste.

### Effect of Thermal Oxidation Pretreatment of PE and PP on ATT Reaction.

**Fig. 3.**
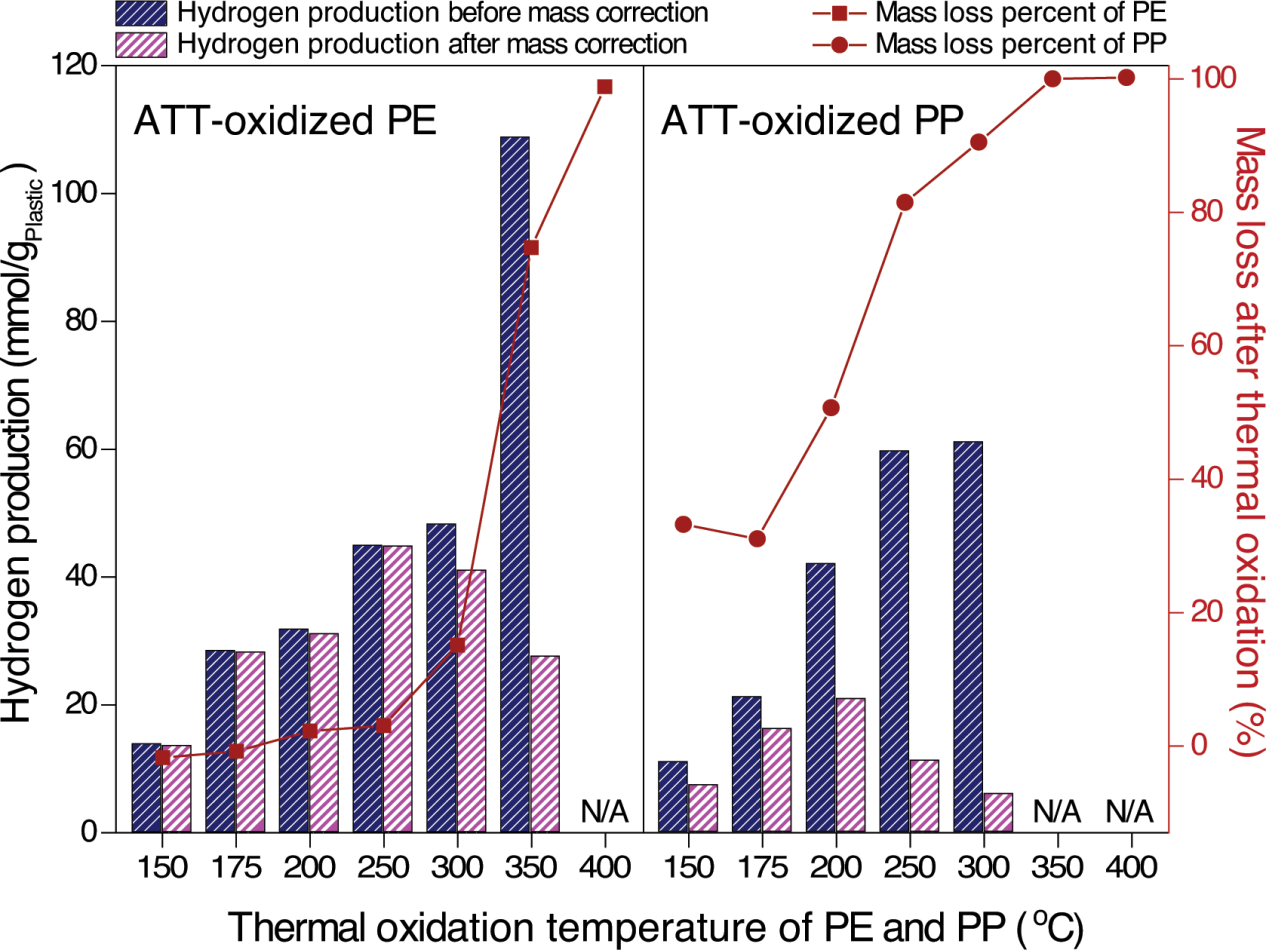
Hydrogen production yields of thermally oxidized PE and PP at various temperatures. The bars represent the H_2_ production yields of oxidized PE and oxidized PP with a NaOH-to-plastic ratio of 5 in ATT, shown both before and after mass correction (blue and pink, respectively). The red line on the right-axis indicates the mass loss percentage as a function of the oxidation temperature. N/A denotes temperatures at which sample collection was not possible due to combustion.

Following oxygen functionalization via thermal oxidation, we investigated how the oxidation temperature influences hydrogen production during ATT. As shown in Fig. 3, the oxidation pretreatment temperature significantly affects hydrogen yields in both PE and PP.

For thermally oxidized PE, hydrogen production progressively increases with oxidation temperature, reaching a maximum of 108.9 mmol H_2_/g_PE_ at 350 °C. This trend is consistent with the higher oxygen incorporation observed at elevated oxidation temperatures as shown in *SI Appendix*, Fig. S3, confirming that increased oxygen-containing functional groups by higher temperature enhances PE reactivity in ATT. However, after accounting the mass loss during thermal oxidation, the effective hydrogen yield at 350 °C decreases from 108.9 to 27.5 mmol H_2_/g_PE_, as PE loses approximately 74% of its mass. When considering mass efficiency, the optimal oxidation temperature for PE is therefore around 250 °C, yielding 44.8 mmol H_2_/g_PE_ after mass correction.

For PP, the hydrogen production trend slightly differs from that of PE. While hydrogen production initially increases with oxidation temperature, the improvement plateaus around 250 °C. This may be due to the lower thermal stability of PP compared to PE, as the presence of tertiary carbon atoms in PP increases the degradation and mass loss at elevated temperatures. At 350 °C, excessive mass loss prevents sample collection due to combustion. The highest hydrogen yield for PP was obtained at 300 °C, reaching 60.9 mmol H_2_/g_PP_. However, after mass correction, the maximum yield was achieved at 200 °C with 20.7 mmol H_2_/g_PP_, emphasizing the trade-off between oxygen incorporation and polymer stability.

These results demonstrate that oxidation pretreatment temperature is a critical parameter governing hydrogen production efficiency of PE and PP. Optimizing the oxidation conditions is essential to maximize hydrogen production yields in ATT, and the most effective temperatures are approximately 250 °C and 200 °C, for PE and PP, respectively when accounting for mass loss. Balancing oxygen incorporation and polymer stability is key to ensuring efficient hydrogen production from plastic waste in ATT processes.

### Theoretical Investigation of ATT Reaction Mechanisms.

**Fig. 4.**
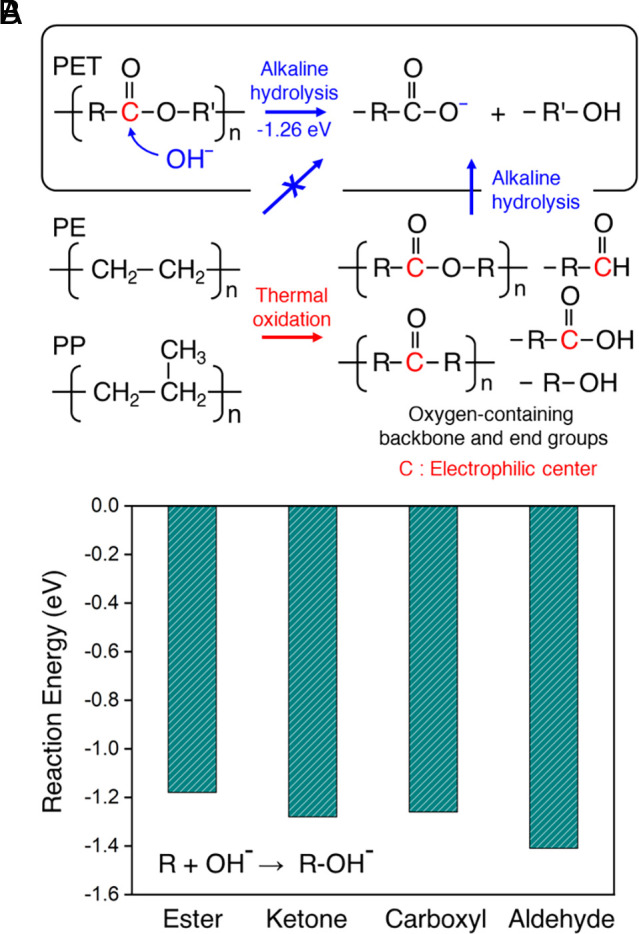
Reaction mechanisms and energetics of plastic waste in alkaline hydrolysis using the first-principles calculations. (*A*) Schematic illustration of alkaline hydrolysis pathways for PET, thermally oxidized PE, and PP. (*B*) Calculated reaction energies for hydroxide ion (OH^−^) addition to various oxygen-containing functional groups formed during thermal oxidation, demonstrating the thermodynamic favorability of these reactions.

To elucidate the molecular-level mechanisms governing the distinct reactivity patterns of PET, PE, and PP in ATT, we performed density functional theory (DFT) calculations to evaluate the energetics of hydroxide ion (OH^−^) in addition to various functional groups. As shown in Fig. 4*A*, ester linkages in PET readily undergo alkaline hydrolysis through nucleophilic attack by OH^−^ at the electrophilic carbonyl carbon, resulting in polymer chain cleavage and the formation of carboxylate and alcohol end groups as seen in *SI Appendix*, Fig. S6. This process is thermodynamically favorable, with a reaction energy of −1.26 eV, demonstrating that PET can directly participate in ATT reactions. In contrast, PE and PP, which consist exclusively of saturated hydrocarbon chains, lack viable reaction pathways for OH^−^ addition. Our calculations indicate that direct nucleophilic attack on saturated C–H or C–C bonds is thermodynamically prohibited due to the electron-rich nature of these bonds and steric hindrance. This explains why pristine PE and PP exhibit negligible reactivity in ATT. However, thermal oxidation introduces oxygen-containing functional groups into the PE and PP backbone and chain ends, creating reactive sites for subsequent alkaline hydrolysis (55).

Our DFT calculations revealed that all major oxidation products, esters (-COO-), ketones (-CO-), carboxylic acids (-COOH), and aldehydes (-CHO), can form stable adducts with OH^−^, with reaction energies ranging from −1.18 to −1.41 eV, as shown in Fig. 4*B*. Among these, aldehyde groups exhibited the highest thermodynamic driving force for OH^−^ addition (−1.41 eV), followed by ketone (−1.28 eV), carboxyl (−1.26 eV), and ester groups (−1.18 eV). This thermodynamic favorability arises from the electron-withdrawing nature of the oxygen-containing groups, which creates electrophilic carbon centers susceptible to nucleophilic attack, similar to native ester linkages in PET. Notably, our calculations show that alcohol groups (-OH), which may form during oxidation or as reaction intermediates, are unreactive toward OH^−^ addition, as shown in *SI Appendix*, Fig. S5. This is because the C–OH bond lacks an electrophilic carbon center, and the electronegative oxygen increases the electron density at the adjacent carbon through inductive effects, making it resistant to nucleophilic attack. This phenomenon partly explains the reduction in high-temperature hydrogen production for ATT in PET. In contrast, carbonyl-containing groups (C=O) possess π-bonding, which creates an electron-deficient carbon center, rendering them highly susceptible to OH^−^ addition.

These theoretical insights highlight the necessity of thermal oxidation pretreatment to enhance the reactivity of ATT in PE and PP. The oxidation process transforms the unreactive hydrocarbons into oxygen-functionalized polymers, rendering them suitable for alkaline hydrolysis. Furthermore, our calculations also suggest that the degree and nature of oxygen incorporation during thermal oxidation directly influences ATT reactivity, with a higher abundance of carbonyl-containing groups facilitating more efficient polymer breakdown and hydrogen production.

### Effect of NaOH-to-Plastic Ratio on ATT Reaction of Thermally Oxidized PE and PP.

**Fig. 5.**
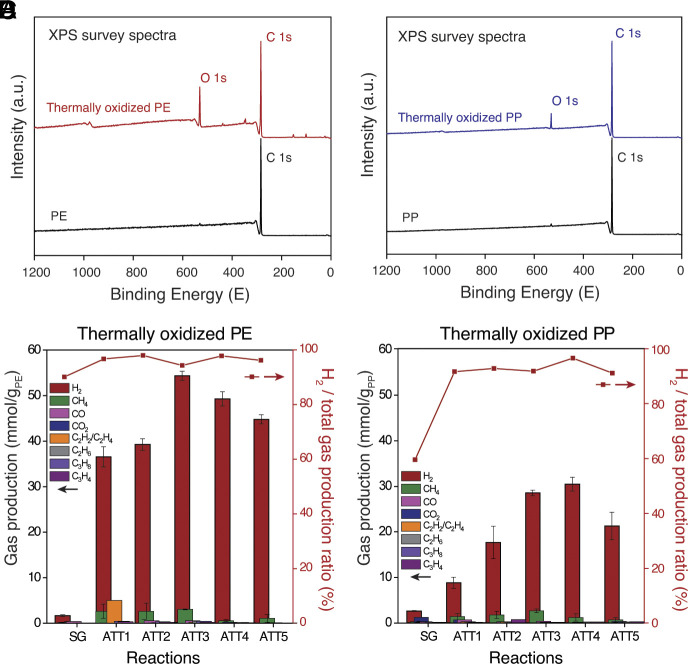
XPS analysis and hydrogen production behavior of thermally oxidized PE and PP under ATT conditions. XPS survey spectra of pristine and thermally oxidized (*A*) PE and (*B*) PP, showing the emergence of the O 1 s peak after oxidation, confirming the incorporation of oxygen-containing functional groups. Gas production results for (*C*) thermally oxidized PE and (*D*) thermally oxidized PP under SG and ATT reactions with different NaOH-to-plastic ratios. The bar chart (left-axis) represents the amounts of various gaseous products after mass correction, while the red line (right-axis) indicates the H_2_-to-total gas production ratio. Error bars represent the SD of three independent samples.

After optimizing the thermal oxidation temperatures of PE (250 °C) and PP (200 °C), XPS analyses were conducted to verify surface oxidation of PE and PP (Fig. 5 *A* and *B*). The XPS spectra clearly shows the emergence of an O 1 s peak in thermally oxidized samples compared to pristine PE and PP, confirming the incorporation of oxygen-containing functional groups.

Then, we systematically investigated the effect of the NaOH-to-plastic ratio on the gas production during the SG and ATT reactions. As shown in Fig. 5 *C* and *D*, both PE and PP exhibited significantly higher total gas and H_2_ production in ATT compared to SG, indicating that NaOH addition in ATT enhances polymer decomposition and H_2_ production. Furthermore, across all NaOH ratios (ATT1–ATT5), thermally oxidized PE and PP produced substantially higher H_2_ gas yields than their nonoxidized counterparts, consistent with the enhanced reactivity implied by the XPS results. This trend also aligns with the results observed in ATT-PET reactions, suggesting that oxygen incorporation during thermal oxidation facilitates the reaction with NaOH.

Regarding gas distribution, nonoxidized PE and PP primarily generate H_2_ together with carbon-based products such as CO_2_, CO, and hydrocarbons. However, after thermal oxidation, ATT reaction significantly reduced the formation of these carbon-based products. As shown in Fig. 5 *C* and *D*, the purity of hydrogen gas, represented by the H_2_-to-total gas production ratio, exceeds 94% for oxidized PE and 91% for oxidized PP across all NaOH ratios. This improvement is attributed to the catalytic role of NaOH coupled with the increased oxygen-functionalized surfaces confirmed by XPS, which together promote selective low-temperature hydrogen production while inhibiting carbonaceous byproducts.

Real-time gas production profiles further highlight the role of the NaOH-to-plastic ratio. For thermally oxidized PE (250 °C), hydrogen production begins at lower temperatures (300 to 400 °C), while the production rate in the higher temperature range increases with NaOH loading (*SI Appendix*, Fig. S7). A similar trend is observed for thermally oxidized PP (200 °C), as shown in *SI Appendix*, Fig. S8. Although the onset temperature of hydrogen evolution remains largely unchanged, the gas production at higher temperatures is strongly dependent on the NaOH ratio. The results in Fig. 5, along with the real-time gas evolution behavior in *SI Appendix*, Figs. S7 and S8, underscore the crucial role of the NaOH-to-plastic ratio of thermally oxidized PE and PP during ATT.

This hydrogen production behavior in thermally oxidized PE and PP can be attributed to the distinct reactivity of the oxygen-functionalized regions in the polymer structure. The first hydrogen peak at low temperature likely originates from the decomposition of oxidized polymer segments containing carbonyl and related oxygen-functional groups formed during thermal oxidation and subsequently transformed under alkaline ATT conditions. These functionalized regions act as catalytic sites that lower the activation energy for hydrogen abstraction through transition state stabilization and facilitate hydrogen abstraction at relatively lower temperatures. These catalytic centers enable hydrogen release pathways that are kinetically favored at lower temperatures compared to the purely thermal decomposition mechanisms that dominate at higher temperatures. Following this catalytically facilitated hydrogen release at lower temperatures, the residual polymer fragments undergo significant transformation, resulting in carbon-enriched domains with more condensed structures (e.g., cross-linked networks, and polyconjugated systems). These transformed structures no longer benefit from the catalytic effects of oxygen functionalities that facilitated the initial hydrogen release. Instead, they require more thermal energy to overcome the activation barriers for C-H bond cleavage, accounting for the second hydrogen production peak observed at higher temperatures (500 to 650 °C).

Notably, the optimal NaOH-to-plastic ratio does not strictly adhere to the stoichiometric predictions of ATT but instead appears to have a unique optimal balance specific to each plastic type. This deviation may be attributed to the distinct chemical and physical properties of plastics compared to biomass, which influence their decomposition behavior. During ATT, thermally oxidized PE and PP may react with NaOH through a combination of decomposition, carbonation, and pyrolysis, which significantly affects the heat and mass transfer dynamics within the system. Our findings indicate that at an optimal NaOH-to-plastic ratio, both PE and PP achieve high hydrogen yields while minimizing the formation of carbon-based byproducts such as CO, CO_2_, and hydrocarbons. These results highlight the importance of optimizing the NaOH ratio for efficient ATT reactions, ultimately maximizing hydrogen production while reducing undesirable carbon emissions. By precisely tuning the reaction conditions, ATT provides a viable pathway for converting plastic waste into clean hydrogen energy.

### Distribution of Carbon from Plastics from ATT Reaction.

The fate of carbon derived from plastic subjected to ATT was investigated to evaluate the environmental impacts and efficiency of the process. Results from ATT experiments (Fig. 1 for PET, Fig. 5 for thermally oxidized PE and PP) confirmed the environmental advantages of ATT, notably the negligible atmospheric emission of gaseous CO_2_. However, EA-derived reaction equations (Eqs. **2**–**4**) demonstrated that actual hydrogen yields for each plastic type were lower than the theoretical maximum estimated by stoichiometric calculations, suggesting partial diversion to alternative pathways, such as conventional thermal decomposition.

[2] $$\begin{array}{c} C_{10}H_{8}O_{4}+\;20\;\mathrm{NaOH}\;+\;6\;H_{2}O\to 20\;H_{2}+\;10\;{\mathrm{Na}}_{2}{\mathrm{CO}}_{3}, \end{array}$$

[3] $$\begin{array}{c} C_{6.8}H_{12.8}O_{0.2}+\;13.6\;\mathrm{NaOH}\;+\;6.6\;H_{2}O\\ \to \;17.3\;H_{2}+\;6.3\;{\mathrm{Na}}_{2}{\mathrm{CO}}_{3}, \end{array}$$

[4] $$\begin{array}{c} C_{6.3}H_{10.8}O_{0.8}+\;12.7\;\mathrm{NaOH}\;+\;5.5\;H_{2}O\\ \to \;19.8\;H_{2}+\;6.8\;{\mathrm{Na}}_{2}{\mathrm{CO}}_{3}. \end{array}$$

**Fig. 6.**
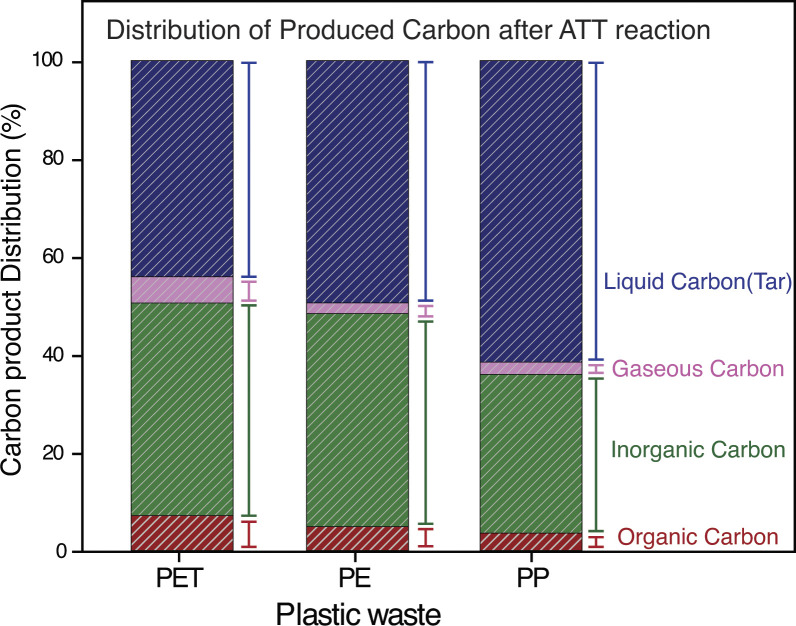
Distribution of carbon-based species from PET, PE, and PP plastics after ATT. Carbon distribution analysis of PET, thermally oxidized PE, and thermally oxidized PP after the ATT reaction. Approximately 32 to 44% of the carbon was converted into inorganic carbon, 44 to 62% into liquid-phase carbon, and less than 13% into gaseous and residual organic carbon species.

The carbon capture and storage (CCS) potential of ATT was further assessed through detailed analyses of carbon transformations and distributions from plastic waste. As illustrated in Fig. 6, post-ATT carbon distributions were approximately 32 to 44% inorganic carbon (Na_2_CO_3_), 44 to 62% liquid-phase carbon (tar and wax), and less than 13% residual gaseous and organic carbon species. Specifically, theoretical hydrogen generation for PET according to Eq. **2** is 58 mmol/g_PET_, but actual production, measured at 20.8 mmol/g_PET_ (Fig. 1), revealed that around 67.4% of Na_2_CO_3_ formation involved captured CO_2_ generated from thermal decomposition. Comparable analyses of thermally oxidized PE (Eq. **3**) and PP (Eq. **4**) showed that 54.7% and 59.2% of Na_2_CO_3_, respectively, originated from captured CO_2_. Consequently, ATT reaction selectivities for PET, PE, and PP were calculated to be approximately 32.7%, 49%, and 41%, respectively, highlighting opportunities for further process optimization. These findings reinforce that post-ATT carbon from plastic waste is predominantly sequestered as Na_2_CO_3_ or remains as tar and wax, with minimal gaseous CO_2_ emissions, underscoring the environmental sustainability of ATT. The environmental significance of these carbon-containing products should, however, be interpreted carefully. The inorganic carbon is mainly present as stable Na_2_CO_3_, and previous studies have shown that NaOH can be regenerated from Na_2_CO_3_ using Ca(OH)_2_, with the carbon permanently fixed as CaCO_3_ (31). Because CaCO_3_ is less soluble, it is more suitable for long-term carbon immobilization. In addition, CaCO_3_ is widely used as an industrial raw material in applications such as cement, paper, plastics, paints, and fillers.

### H_2_ Production of Mixed Plastic Wastes of PET, PE, and PP in ATT Reaction.

After optimizing ATT conditions for individual plastics, we extended our study to mixed plastic waste composed of PET, PE, and PP in a 1:1:1 ratio, simulating common waste streams. The experiments were performed using commercial PET, PE, and PP waste, further reflecting realistic feedstock characteristics. ATT was also applied to commercial polystyrene (PS) waste, and its gas production behavior is shown in *SI Appendix*, Fig. S9.

**Fig. 7.**
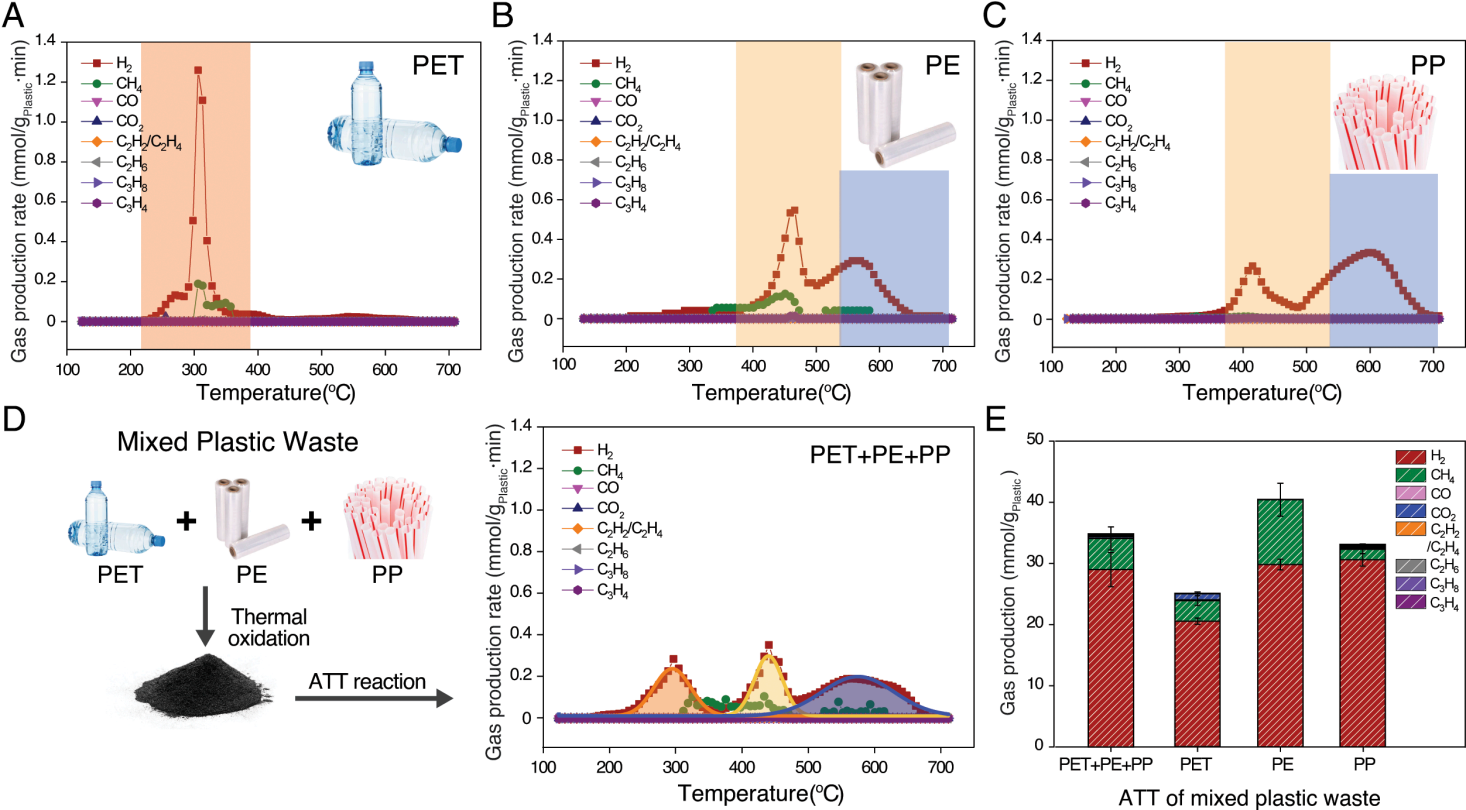
Gas production rates of individual and mixed plastics waste of PET, PE, and PP during ATT reaction. Real-time gas production profiles for the ATT process with a NaOH-to-plastic ratio of 3 within a temperature range of 100 to 700 °C for (*A*) PET after thermal oxidation (200 °C, 50 h), (*B*) PE after thermal oxidation (200 °C, 50 h) and (*C*) PP after thermal oxidation (200 °C, 50 h). (*D*) Schematic illustration of the ATT reaction for thermally oxidized mixed plastics (PET, PE, and PP) with the corresponding gas production profile. (*E*) Comparison of gas product distributions from the ATT of mixed plastics and individual plastics (PET, PE, and PP). Error bars represent the SD of three independent samples.

Fig. 7 presents the gas production profiles of oxidized individual PET, PE, and PP waste, as well as their mixed waste under ATT conditions, showing distinct variations depending on the plastic type and its combination. The decomposition of mixed plastic feedstock is more complex, reflecting the thermal and chemical interactions among its components. The first major hydrogen peak appears at ~297 °C, aligning with the hydrogen production observed in the ATT reaction of thermally oxidized PET (Fig. 7 *A* and *D*). The second and third peaks at 441 °C and 573 °C correspond to the hydrogen evolution temperatures of PE and PP, respectively, as seen in Fig. 7 *B*–*D*. This suggests that each plastic in the mixture follows decomposition pathways similar to those observed for individual plastics. Gaussian fitting analysis (Fig. 7*D*) further supports these results, as the hydrogen peaks closely match those of individual thermally oxidized plastics. Fig. 7*E* quantitatively compares gas production from ATT of mixed plastics with that of PET, PE, and PP waste individually. Notably, H_2_ yields from mixed plastics are comparable to those from individual plastics, demonstrating the potential of ATT for processing mixed plastic waste. However, the influence of nonreactive inorganic fillers present in real and heterogeneous plastic waste streams should not be overlooked when evaluating the apparent H_2_ yield. The high selectivity for hydrogen over carbon-based byproducts underscores the effectiveness of ATT in producing high-purity hydrogen while minimizing unwanted side reactions. Thermal oxidation pretreatment enables efficient hydrogen production over a broad temperature range by leveraging the combined properties of PET, PE, and PP. These findings highlight ATT as a scalable and robust approach for converting mixed plastic waste into valuable hydrogen fuel with no CO_2_ emissions. The ability to process mixed plastics without presorting simplifies recycling, improves efficiency, and maximizes hydrogen production. ATT thus offers a promising solution for sustainable plastic-to-hydrogen conversion, addressing plastic waste challenges while generating clean energy.

### Life Cycle Assessment (LCA) Results of PET, PE, and PP in ATT.

**Fig. 8.**
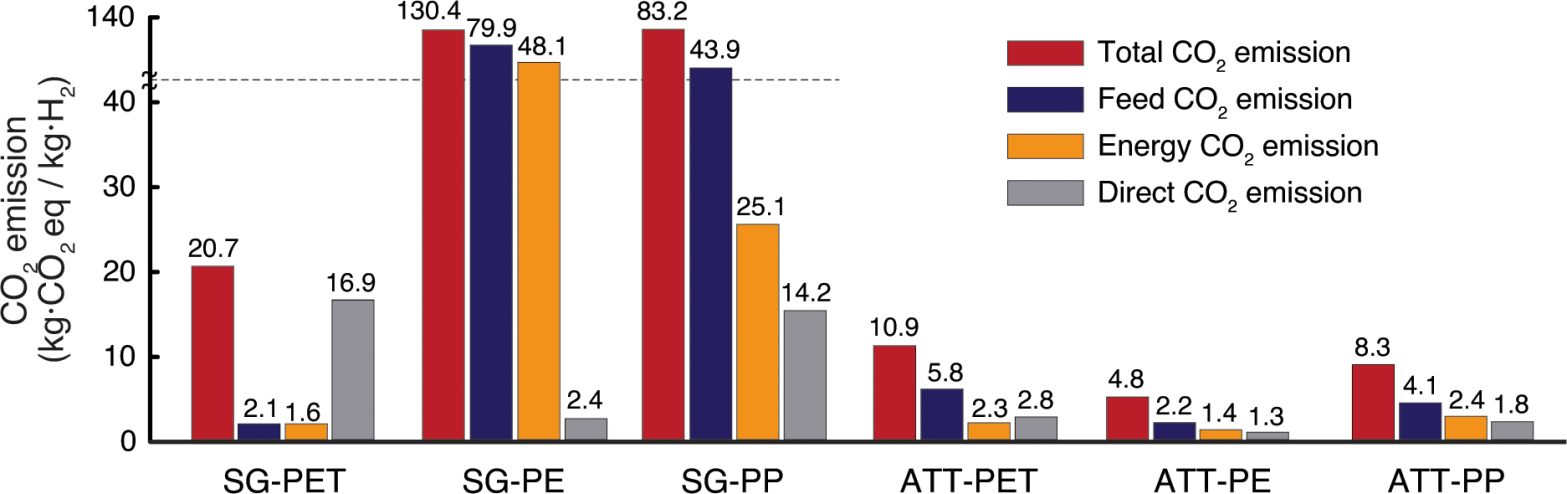
LCA results of PET, PE, and PP in ATT reaction compared to SG reaction. CO_2_ emission breakdown of H_2_ production; i) CO_2_ emission by feedstock, ii) CO_2_ emission by energy, and iii) direct CO_2_ emission.

The core LCA of H_2_ production was conducted through process simulation within the defined LCA boundary (*SI Appendix*, Fig. S10). The CO_2_ emission results for the PET, PE, and PP cases in ATT3, together with those for SG-PET, PE, and PP, are presented in Fig. 8. Their principal sources of CO_2_ emissions were identified. Emissions arise from the feedstock input: In SG, only steam is considered, whereas in ATT, the NaOH input is accounted for. Additional energy is required both to raise the polymer to the reaction temperature and to supply steam, which involves sensible and latent heat. Further heat energy is needed to drive the enthalpy change associated with the highly endothermic reaction that produces H_2_, CO_2_, and other hydrocarbons. Finally, assuming that all gases generated in the reactor except H_2_, are combusted in the furnace as a heat source, the direct CO_2_ released to the atmosphere is included in the assessment.

In the SG-PET case, the emissions amounted to 20.67 kg-CO_2_ eq/kg-H_2_, with the largest contribution coming from direct CO_2_ (16.96 kg-CO_2_ eq/kg-H_2_). In other words, relative to the amount of H_2_ produced, the generation of CO_2_ and other hydrocarbons was disproportionately high, resulting in elevated overall emissions. In contrast, the SG-PE and SG-PP cases showed significantly lower H_2_ yields compared to SG-PET, which resulted in very high total CO_2_ emissions. These processes therefore cannot be considered viable routes for targeted H_2_ production. In the ATT process, the supply of NaOH slightly increased the feedstock-related CO_2_ emissions. However, when the CO_2_ emission factor of the resulting Na_2_CO_3_ was considered, the overall emissions were comparable to those of the SG process. Notably, in the PE case under ATT, the H_2_ yield was very high, which led to the lowest CO_2_ emissions observed (4.83 kg-CO_2_ eq/kg-H_2_). The ATT-PET and ATT-PP cases also showed lower CO_2_ emissions than SG-PET, with values of 10.90 and 8.33 kg-CO_2_ eq/kg-H_2_, respectively. Such reductions can be attributed to the markedly lower direct CO_2_ emission (i.e., CO_2_ emitted directly into the atmosphere) compared with SG. Experimental results confirmed that during the ATT reaction, most of the supplied NaOH was converted into Na_2_CO_3_, and this transformation was the key factor underlying the reduced direct CO_2_ emissions.

For the most widely commercialized hydrogen production route, steam-methane reforming, it is well established that in the absence of a CO_2_ capture unit, the process results in approximately 8 to 10 kg-CO_2_ eq/kg-H_2_ (56). In comparison, coal-based steam gasification has been reported to emit around 19.37 to 22.87 kg-CO_2_ eq/kg-H_2_ (57). In our SG process, achieving a higher H_2_ yield requires operation at elevated temperatures. However, extremely high reaction temperatures inevitably cause greater heat losses, increased energy demand, and substantial direct CO_2_ emissions, indicating that even an optimized configuration would still result in relatively high CO_2_ emissions. The ATT reaction we evaluated also presents opportunities for modifying operating conditions to realize greater environmental benefits. Potential strategies include utilizing undesired gases (primarily hydrocarbons) as fuel, implementing NaOH recycling, and optimizing reaction temperature. Exploring these economic–environmental trade-offs will be an important direction for future work in assessing whether ATT could serve as a viable alternative to the commercial processes. Nevertheless, even with the relatively simplified LCA employed in this study, the significant reduction in direct CO_2_ emissions suggests that ATT-based hydrogen production may provide environmental competitiveness relative to conventional processes.

## Summary

This study successfully demonstrated the potential of ATT as a highly effective and scalable approach for converting plastic waste, including mixed plastic waste, into clean hydrogen energy. Optimizing key reaction parameters such as the NaOH-to-plastic ratio and thermal oxidation pretreatment conditions enhanced hydrogen production at relatively low temperatures, outperforming conventional gasification while minimizing carbon-based byproducts. Thermal oxidation introduced oxygen-containing functional groups into PE and PP, significantly improving their reactivity in ATT, facilitating efficient polymer decomposition at lower temperatures, and ultimately increasing hydrogen yield. These findings highlight the distinct advantage of ATT in achieving efficient hydrogen generation at substantially lower temperatures while effectively suppressing carbon-based byproducts such as CO_2_ compared to conventional gasification processes.

Moreover, ATT effectively converts mixed plastic waste into hydrogen without complex polymer separation, demonstrating its practical feasibility for large-scale plastic waste management. The direct transformation of PET, PE, and PP mixtures into high-purity hydrogen underscores their potential as viable and scalable solutions for sustainable plastic-to-hydrogen conversion. This study establishes ATT as a promising strategy for addressing plastic waste challenges while simultaneously advancing clean energy production, paving the way for future research and industrial implementation.

## Materials and Methods

### Materials.

PET ((C_10_H_8_O_4_)_n_, CAS. 25038-59-9)), PE [H(CH_2_CH_2_)_n_H, CAS. 9002-88-4)], and PP ([CH_2_CH(CH_3_)]_n_, CAS. 9003-07-0) were purchased from Sigma Aldrich. Waste plastic samples, including PET, PE, PP, and PS, were prepared from commercially available plastic products purchased from a convenience store, including actual water bottles (Samdasoo, South Korea). The collected samples were washed, dried, cut into pieces smaller than 0.5 cm, and then ground into powder with a particle size of approximately 200 μm using a laboratory blender (Wonder Blender, WB-1). NaOH (98.8% purity) for alkaline treatment was purchased from Daejung Chemicals & Metals Co., Ltd.

### Reactor System.

The reactor system is composed of a three-zone tubular furnace and a quartz tube of 2.54 cm outer diameter and 100 cm length (*SI Appendix*, Fig. S11). An alumina boat (3 mL) containing the appropriate amount of plastics and alkali was placed inside a quartz tube, positioned in the heating zone of the furnace. Real-time temperature monitoring during the reactions was facilitated by a thermocouple installed inside the quartz tube of the reactor, recording the temperature. The reactor is also connected to a gas chromatograph (GC, Agilent, micro-GC Agilent 490) for real-time analysis of the generated gaseous products. Moisture and heavy hydrocarbons were removed from the products using a condenser and liquid trap before they were collected in GC.

### ATT Reactions.

The experiments were conducted to analyze the differences in SG and ATT results for oxygen-containing PET and C–C backbone polymers composed solely of carbon and hydrogen (PE, PP). For each experiment, 0.05 g of the polymer was mixed with a 50 wt.% NaOH solution, which was subsequently inserted into the reactor. A mass flow controller (MFC) was used to supply N_2_ carrier gas at a rate of 50 mL min^−1^ through a tube connected to the quartz tube of the reactor. After placing the sample in the center of the reactor, the system was purged with N_2_ gas for over 30 min before each experiment. The furnace temperature was raised from room temperature to 100 °C for over 20 min and maintained for 10 min to preheat the sample. During the reaction, water was fed by a syringe pump equipped with an 18 G needle at a flow rate of 23 μL min^−1^. The injected water was passed through a 5 m long, 1/8 in. tube placed inside a 150 °C heating box, where it was vaporized, and the resulting steam was introduced into the reactor by N_2_ carrier gas. Under a steam environment, the reactor temperature was increased from 100 to 700 °C at a rate of 2 °C min^−1^ and then maintained until the reaction was completed. The gas products were monitored in real-time using a micro-GC and collected in a gas Tedlar bag throughout the reaction.

For PE and PP, C–C backbone polymers, thermal oxidation pretreatment was performed in an air environment using a furnace at 150 to 350 °C for 50 h. For thermally oxidized PE and PP, the hydrogen production values before and after mass correction were calculated based on two different mass references. During thermal oxidation, both PE and PP undergo partial mass loss, and this mass loss becomes larger as the oxidation temperature increases. To quantify this effect, we defined the mass loss percent based on the difference between the initial mass of the plastic before thermal oxidation and the remaining mass after thermal oxidation. Accordingly, the hydrogen production before mass correction is defined as the amount of hydrogen produced per gram of the thermally oxidized plastic used for the subsequent ATT reaction. In contrast, the hydrogen production after mass correction is defined as the amount of hydrogen produced per gram of the original plastic before thermal oxidation. The detailed calculation procedures, including the definitions of mass loss percent and the two normalized hydrogen production values, are provided in *SI Appendix*, section S1. The presence of oxygen in the thermally oxidized polymers was confirmed through FT-IR (Agilent, Cary 360) and elemental analysis (EA, Thermo Flash 2000). For each ATT reaction, 0.05 g of plastic was mixed with NaOH and H_2_O according to the mass ratios based on the ATT equation, considering the C, H, and O ratios.

### Product Analysis.

Throughout the ATT reaction, both gaseous products and solid residues (comprising organic and inorganic carbon) were generated. The gas products were monitored in real time using a micro-GC (Agilent 490) equipped with a micro-thermal conductivity detector (μ-TCD) and two columns. An RT-Molsieve 5A column was used for the analysis of H_2_, N_2_, O_2_, CH_4_, and CO, with Ar as the carrier gas, while a PoraPLOT U (RT-U-bond) column was used for the analysis of CO_2_, C_2_H_4_, C_2_H_6_, C_2_H_2_, C_3_H_8_, and C_3_H_4_, with He as the carrier gas. Both columns were operated at 80 °C. In parallel with the real-time analysis, the total gaseous products generated over the entire reaction period were collected in a Tedlar bag, and the collected gas was subsequently analyzed using the same micro-GC system to determine the total amount of gas produced. The carbon content in the dried residual solids was analyzed using a total organic carbon (TOC) solid sample measurement system (Shimadzu TOC-L equipped with an SSM-5000A solid sample module). In TOC analysis, total carbon (TC) was first measured for the dried solid sample. Inorganic carbon (IC) was then determined by acidification with phosphoric acid at 200 °C under sparging conditions, during which inorganic carbon species were released as CO_2_ and quantified by a nondispersive infrared (NDIR) detector in the TOC analyzer. Organic carbon (OC) was calculated from the difference between TC and IC. The total carbon content was also evaluated from the CO_2_ evolved during combustion of the residual solid at 900 °C. Assuming that all measured IC was present as Na_2_CO_3_, the amount of Na_2_CO_3_ in the residual solid was calculated from the IC value, and the amount of organic carbon was determined from the OC value. Based on these results, the carbon mass balance was established.

FT-IR spectra were recorded using an Agilent Cary 360 FT-IR spectrometer equipped with an ATR accessory. The spectra were collected in the range of 4,000 to 650 cm^−1^ at a resolution of 4 cm^−1^ using 32 scans per sample. Background correction was performed before each measurement. Dried polymer samples were placed directly on the ATR crystal, and all spectra were processed using the same baseline correction procedure. The spectra were compared before and after thermal oxidation to identify the formation of oxygen-containing functional groups.

The thermally oxidized PE and PP were subjected to elemental analysis for C, H, O, and N using a Thermo Flash 2000 elemental analyzer. In the instrument, the samples were oxidatively decomposed at high temperature (1,800 °C) to form gaseous oxidation products, which were subsequently passed through a reduction column to remove excess oxygen. The gas mixture was then separated by gas chromatography and quantified using a thermal conductivity detector. The measured elemental compositions were used for quantitative compositional analysis and for stoichiometric calculations related to the ATT process.

### Density Functional Theory Calculations.

All quantum chemical calculations were performed using Gaussian 16 software package (58). The reactant and product structures were fully optimized using DFT with the B3LYP hybrid functional and the 6-311++G** basis set (59, 60). The ultrafine integration grid was employed for numerical integrations. The SCF convergence threshold was set to 10^−6^ Ha, and geometry optimizations were converged with thresholds for maximum force of 3 × 10^−4^ hartree/bohr. The reaction energies (ΔE) for the addition of OH^-^ to various functional groups were calculated as the difference between the total electronic energies of the products and reactants:

$$\Delta E\;=\;E\left(R-\mathrm{OH}\right)\;-\;\left[E\left(R\right)\;+\;E\left({\mathrm{OH}}^{-}\right)\right],$$

where E(R-OH) is the energy of the OH adduct product, E(R) is the energy of the reactant molecule, and E(OH^−^) is the energy of the hydroxide ion. The optimized Cartesian coordinates of product molecules are provided in *SI Appendix*, Table S1.

### LCA.

The traditional LCA framework is based on the International Standards Organization (61). In this study, an LCA was performed to assess the environmental impacts of SG/ATT processes based on ISO 14040 standard (62). We adopt the gate-to-gate boundary commonly used in the H_2_ production. More importantly, the global warming impacts (GWIs) of the proposed process were compared. GWI is an all-encompassing, expedient global warming metric calculated as accumulated greenhouse gas emissions, expressed as CO_2_ equivalents (CO_2_ eq), by weighing global warming potential. The primary factors influencing GWI include electricity, steam, and direct CO_2_ emissions.

The LCA was conducted by directly incorporating the empirical data obtained from our experimental measurements to ensure high fidelity in the environmental impact analysis. The inventory for the simulation was constructed based on the precise quantification of hydrogen yield, carbon input, and cumulative energy consumption (e.g., reactor heating and maintenance) recorded during the ATT process. To provide a comprehensive environmental profile, the boundary was extended to include the end-of-life stage of by-products, specifically accounting for the incineration of residual components. All greenhouse gas emissions were normalized to a functional unit of 1 kg of H_2_ produced. The specific emission factors and the detailed life cycle inventory database, including the data sources from ecoinvent and openLCA, are systematically documented in *SI Appendix*, Table S2 (63).

## Supplementary Material

Appendix 01 (PDF)

## Data Availability

Study data are included in the article and/or *SI Appendix*.
