# Supplementary material for: Selective and direct hydrogen generation from mixed plastic waste via alkaline thermal treatment with inherent carbon storage
Source: Proc Natl Acad Sci U S A. 2026 Jul 6;123(28):e2537552123. doi: 10.1073/pnas.2537552123 (PMC13367845; doi:10.1073/pnas.2537552123)
Supplement: Supplementary file 1 — Appendix 01 (PDF) [file pnas.2537552123.sapp.pdf]

Supporting Information

Selective and Direct Hydrogen Generation from Mixed Plastic Waste *via* Alkaline Thermal Treatment with Inherent Carbon Storage

Jieun Park[1,†], Hyunah Kim[2,†], Hyerin Seo[1], Jiwon Lee[1], Hyung-Kyu Lim[3], Wonho Jung[4], Ah-Hyung Alissa Park[5,*] and Woo-Jae Kim[1,6,*]

[1] Department of Chemical Engineering and Materials Science, Graduate Program in System Health Science and Engineering, Ewha Womans University, Seoul 03760, Republic of Korea

[2] Department of Materials Science and Engineering, Korea Aerospace University, Gyeonggi-do 10540, Republic of Korea

[3] Division of Chemical Engineering and Bioengineering, Kangwon National University, Chunchenon, Gangwon-do 24341, South Korea

[4] C1 gas refinery R&D center, Sogang University, Seoul 04107, Republic of Korea.

[5] Department of Chemical and Biomolecular Engineering, UCLA Samueli School of Engineering, CA 90095, USA

[6] Institute for Multiscale Matter and Systems (IMMS), Ewha Womans University, Seoul 03760, Republic of Korea

[†] **These authors contributed equally to this work**

Corresponding Authors

Woo-Jae Kim (wjkim1974@ewha.ac.kr)

Ah-Hyung Alissa Park (apark@seas.ucla.edu)

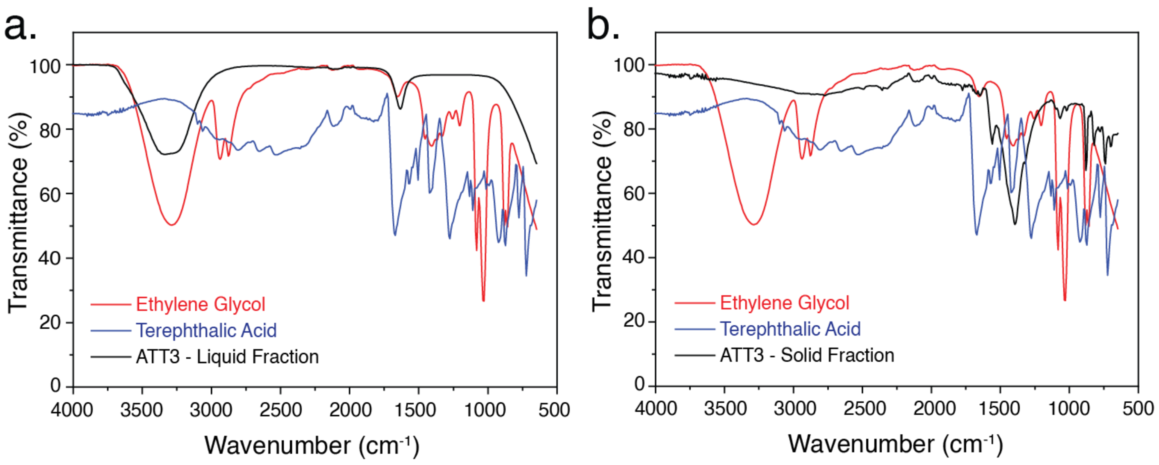

Fig. S1. FT-IR spectra of the products. (a) FT-IR of the liquid sample collected in the downstream-trap and (b) the solid sample obtained from PET after the ATT reaction up to 250 °C. Ethylene glycol (red) and terephthalic acid (blue) were included as reference samples for comparison.

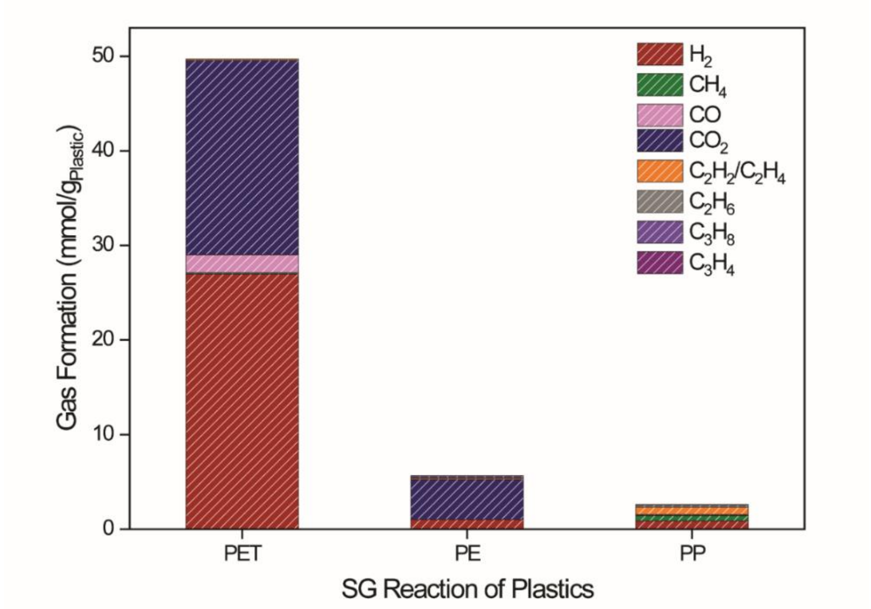

Fig. S2. Gas formation *via* the SG reaction of PET, PE, and PP. Comparison of gas production from the SG process of PET, PE, and PP. The production amounts of H_2, CH_4, CO_2, CO, C_2H_2/C_2H_4, C_2H_6, C_3H_6, and C_3H_4 are shown.

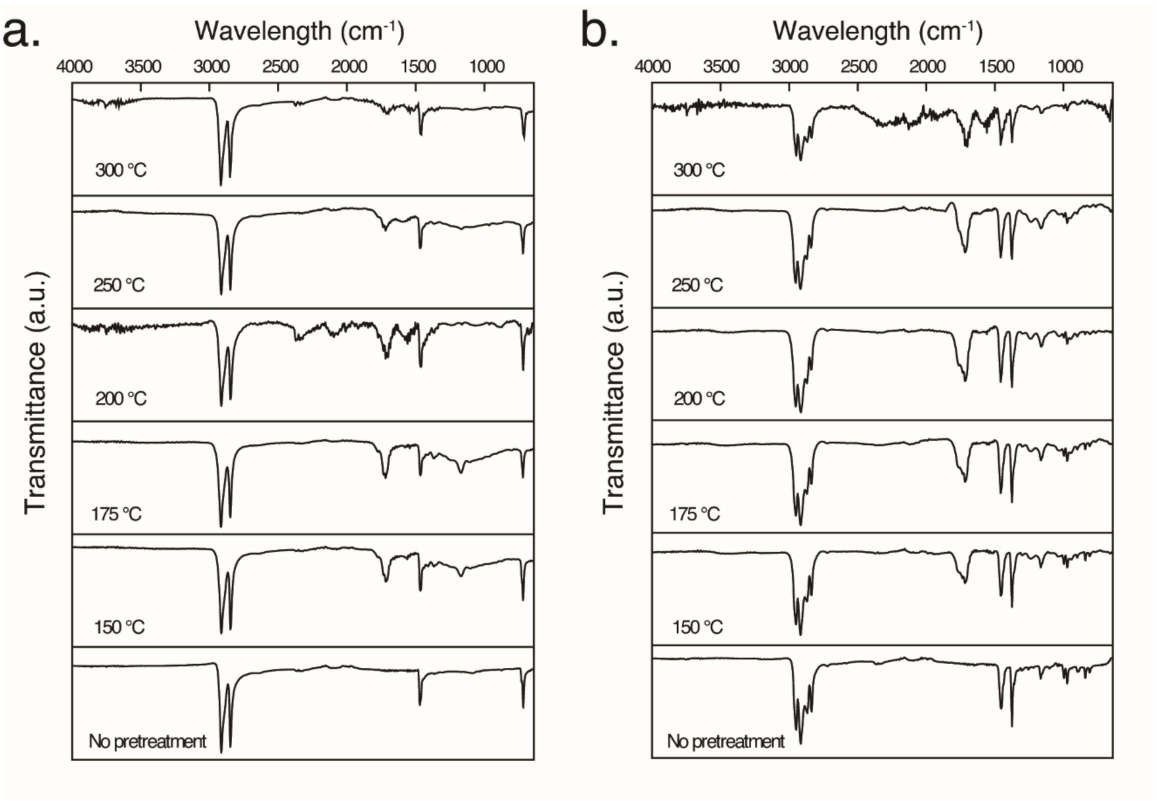

Fig. S3. FT-IR analysis of thermally oxidized PE and PP. FT-IR spectra of (a) PE and (b) PP after thermal oxidation at different temperatures. The spectra reveal the formation of oxygen-containing functional groups, as indicated by the appearance of new peaks, demonstrating the structural modifications induced by thermal oxidation.

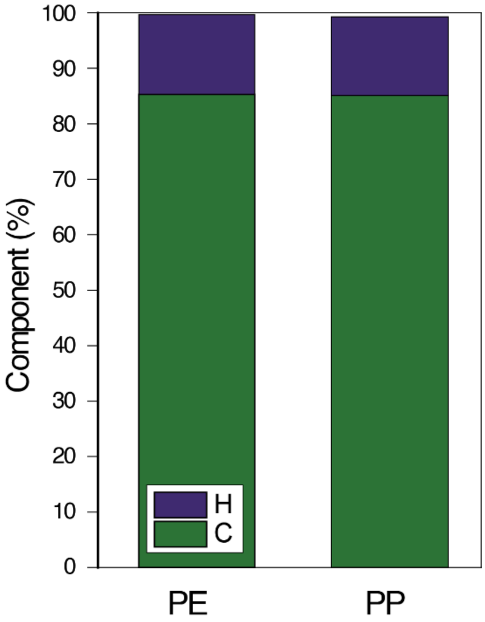

Fig. S4. Elemental analysis of polymer compositions. The elemental composition of (a) PE and (b) PP, including carbon (C) and hydrogen (H) components.

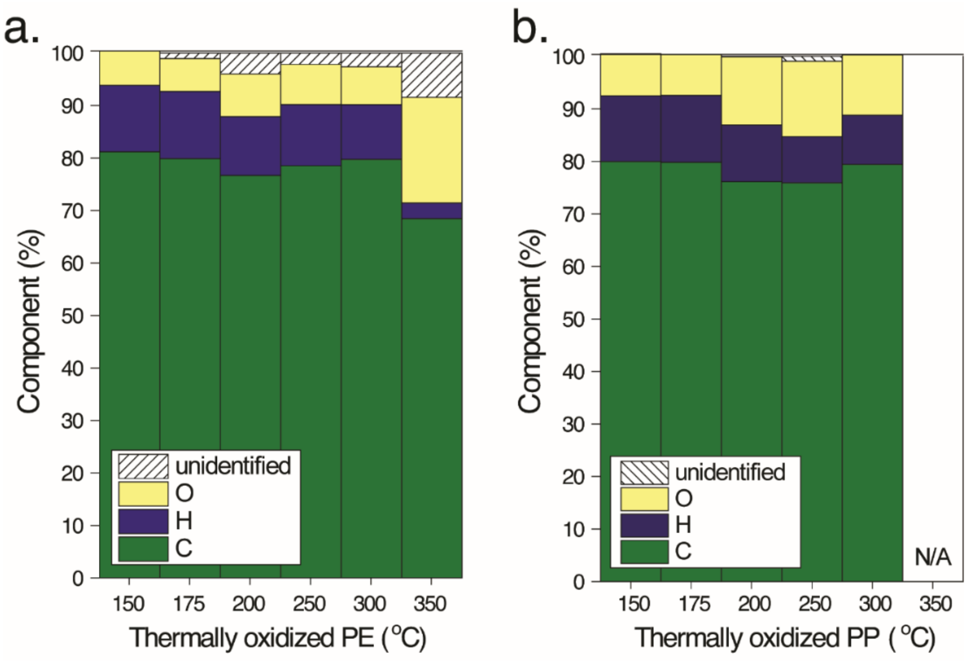

Fig. S5. Elemental analysis of thermally oxidized PE and PP at various temperatures and reaction durations. Effect of thermal oxidation on PE and PP at different temperatures. The thermal oxidation was conducted for 50 h for all samples. The elemental composition, including carbon (C), hydrogen (H), oxygen (O), and unidentified components, is shown as a function of oxidation temperatures. N/A indicates temperatures at which sample collection was not possible due to combustion.

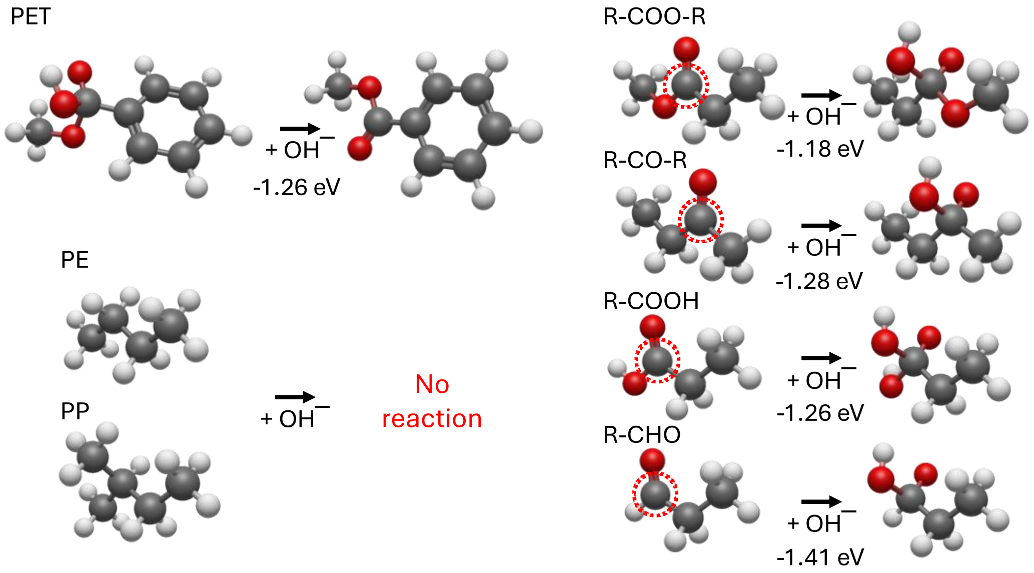

Fig. S6. DFT-optimized molecular structures and reaction energetics for hydroxide (OH^-) addition to various functional groups. DFT calculated structures illustrating the reactivity of OH^- with various oxygen-containing functional groups. The structures highlight the molecular configurations before and after OH^- addition, indicating the reactive sites.

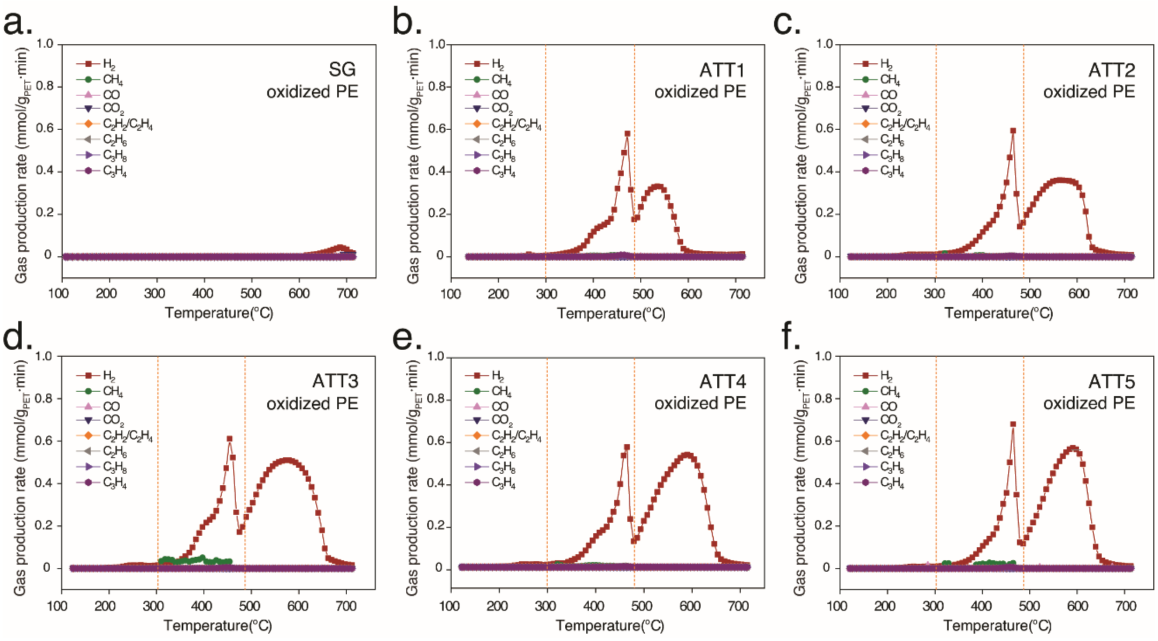

Fig. S7. Gas production rates of thermally oxidized PE during SG and ATT reactions. Real-time gas generation of thermally oxidized PE at 250 °C during (a) SG and (b-f) ATT. Gas production rate is shown for ATT with varying NaOH-to-PE mass ratios, including (b) 1:1 (PE-ATT), (c) 2:1 (PE-ATT2), (d) 3:1 (PE-ATT3), (e) 4:1 (PE-ATT4), and (f) 5:1 (PE-ATT5).

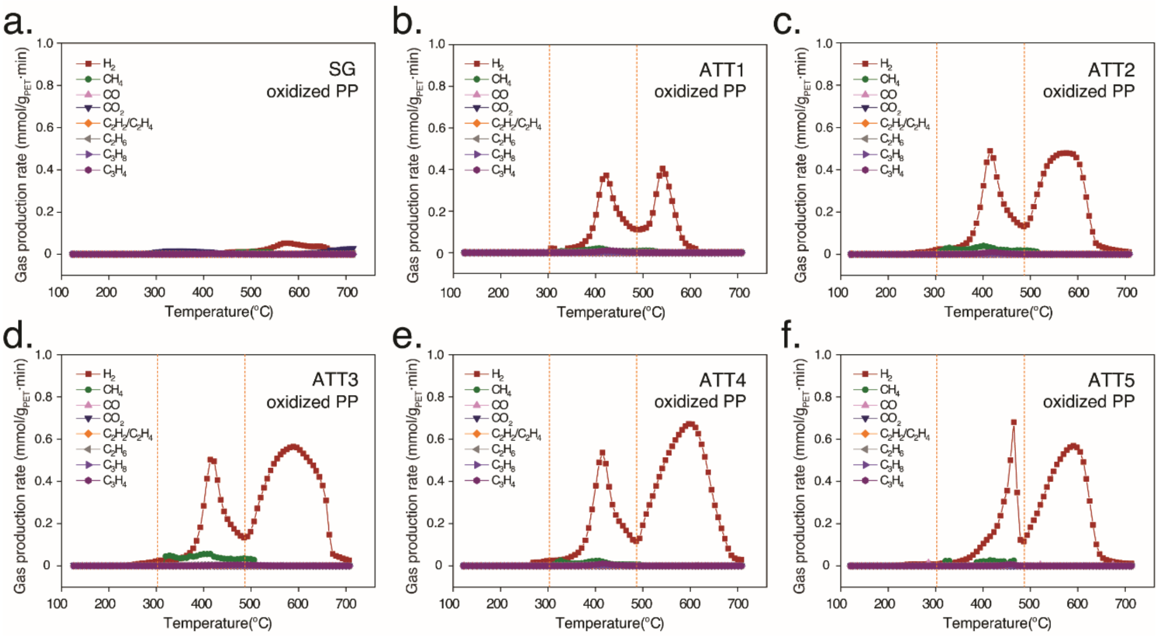

Figure S8. Gas production rates of thermally oxidized PP during SG and ATT reactions Real-time gas generation of thermally oxidized PP at 200 °C during (a) SG and (b-f) ATT. Gas production rate is shown for ATT with varying NaOH-to-PE mass ratios, including (b) 1:1 (PP-ATT), (c) 2:1 (PP -ATT2), (d) 3:1 (PP -ATT3), (e) 4:1 (PP -ATT4), and (f) 5:1 (PP -ATT5).

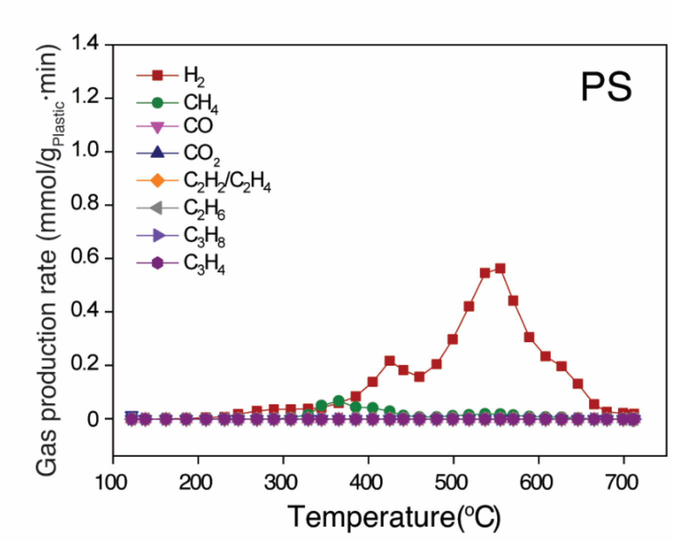

Figure S9. Gas production rates of thermally oxidized waste polystyrene (PS) during ATT3 reaction. Real-time gas production profile of ATT3 reaction within a temperature range of 100 °C to 700 °C for PS after thermal oxidation (200 °C, 50 h).

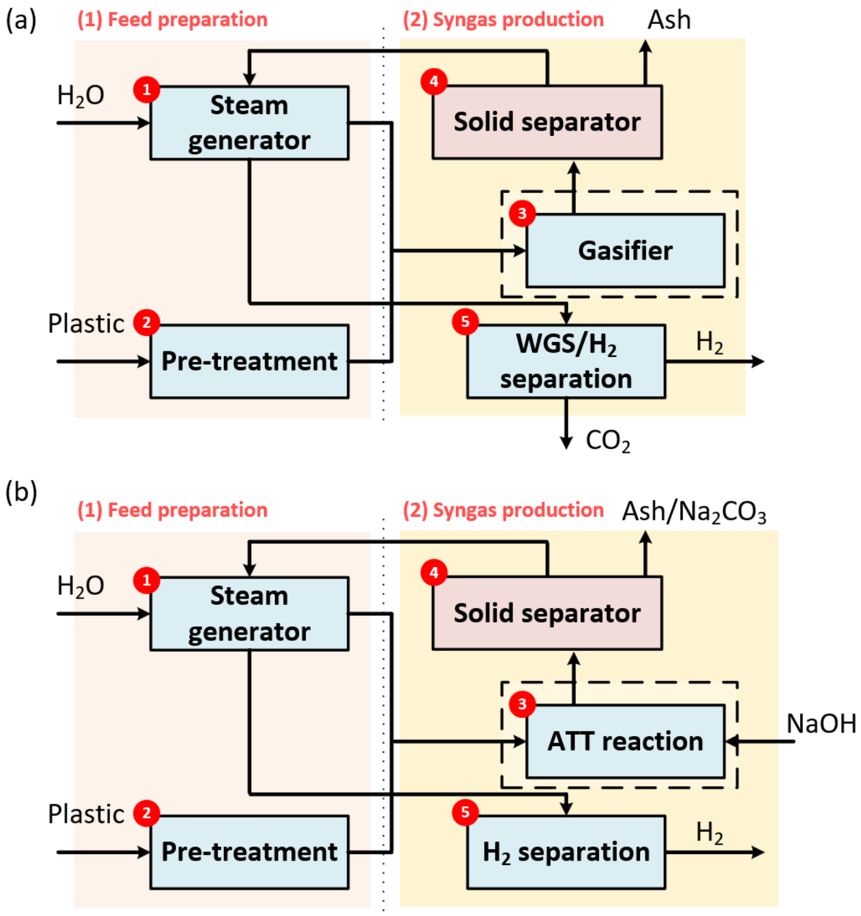

Fig. S10. Process block diagram of (a) SG and (b) ATT for H_2 production. The system boundary includes oxidation and pretreatment of feedstock to produce final high purity H_2.

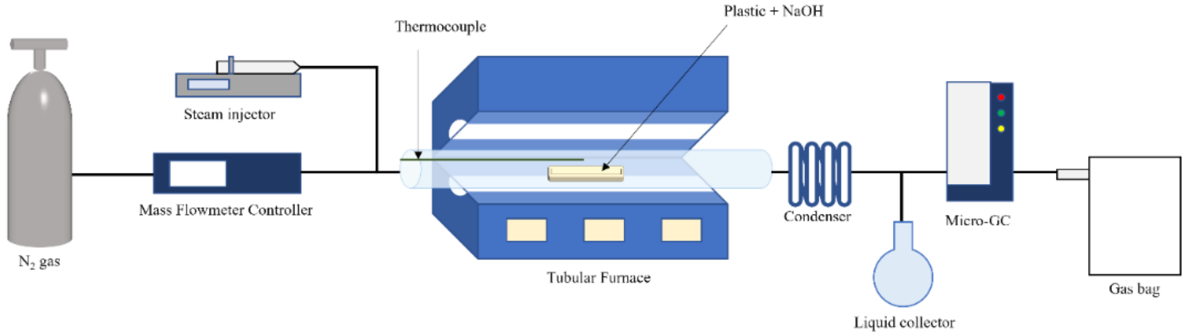

Fig. S11. An illustration of our reactor designed for *in-situ* measurements containing a mass flowmeter with a steam injector, tubular furnace with the thermocouple, and gas chromatograph compartment.

Section S1. Gas Product Quantification: Calculation Procedures

S1.1 Instrument and Nature of Raw Data

Gas products were analyzed using an Agilent 490 Micro-GC equipped with a micro-machined thermal conductivity detector (μ-TCD). Module A (RT-Molsieve 5A column, Ar carrier gas) analyzed H_2, O_2, N_2, CH_4, and CO; Module B (PoraPLOT U (RT-U-bond) column, He carrier gas) analyzed CO_2 and light hydrocarbons. The TCD produces a voltage signal (mV) integrated over time to give a peak area (arbitrary units, a.u.) — the true raw output. The software converts peak areas into concentrations in mole percent (mol%) using pre-determined Response Factors (RF) from calibration (S1.2). For ideal gases, mol% equals vol% numerically (Dalton's law). Detection limit: ~0.5–2 ppmv (1 mol% = 10,000 ppmv).

S1.2 Calibration and Response Factors

Prior to sample analysis, the instrument was calibrated using a certified multi-component standard gas (supplied by Dong-A Specialty Gases) with gravimetrically certified concentrations (mol%) for all target components. The Response Factor for each component *i* is defined as:

$$RF_i = Peak\,Area_i \;/\; C_{i,cal} \quad \text{(Eq. S1)}$$

where $C_{i,cal}$ is the certified concentration of component *i* in the calibration gas (mol%). For an unknown sample: C_i (mol%) = $Peak\,Area_i / RF_i$ (Eq. S2). Calibration was verified periodically with a check gas.

S1.3 Step-by-Step Calculation Procedure

Gaseous products were swept into a gas-tight collection bag using N_2 as an inert carrier at calibrated flow rate F (mL min^{-1}) for reaction time t (min). N_2 serves as an internal standard whose total moles are independently known from F and t, enabling quantification of all other product gases.

Step 1 — Average concentration from replicate GC injections. Each gas bag was injected n times (typically $n = 5$) and averaged: $\bar{C}_i = (1/n)\, \Sigma\, C_{i,k}$ (mol%) (Eq. S3).

Step 2 — N_2 correction for adventitious air. Detected O_2 from air ingress is used to remove the corresponding adventitious N_2 (N_2/O_2 = 78.09/20.95 in dry air): $\bar{C}_{N2,corr} = \bar{C}_{N2} - \bar{C}_{O2} \times (78.09/20.95)$ (Eq. S4).

Step 3 — Mole fraction normalization. After N_2 correction, corrected concentrations are normalized: $x_i = \bar{C}_{i,corr} / \Sigma\bar{C}_{j,corr}$ (Eq. S5). This is unit-independent (mol% or ppmv cancel equally in the ratio).

Step 4 — Moles of N_2 delivered. Using the ideal gas law: $n_{N2} = F \times t / 22.4$ (mmol) (Eq. S6), where F is measured by a calibrated mass flow controller (MFC).

Step 5 — Moles of each product gas. Using N_2 as the internal standard: $n_i = (x_i / x_{N2}) \times n_{N2}$ (mmol) (Eq. S7). The concentration ratio cancels units, so Eq. S7 holds whether concentrations are in mol% or ppmv.

Step 6 — H_2 production normalized to plastic mass (before and after mass correction). During thermal oxidation, PE and PP undergo partial mass loss that increases with oxidation temperature. To account for this, H_2 production is reported using two different mass references. The mass loss from thermal oxidation is first quantified as:

$$\text{Mass loss (\%)} = (m_{initial} - m_{ox}) / m_{initial} \times 100 \quad \text{(Eq. S8)}$$

where $m_{initial}$ is the mass of the original plastic before thermal oxidation (g), and m_{ox} is the remaining mass after thermal oxidation (g), i.e., the mass of the sample actually charged to the ATT reactor. Two normalized H_2 production values are then defined:

$$Y_{before} = n_{H2} / m_{ox} \quad (\text{mmol g}^{-1}) \quad \text{(Eq. S9)}$$

$$Y_{after} = n_{H2} / m_{initial} \quad (\text{mmol g}^{-1}) \quad \text{(Eq. S10)}$$

Y_{before} represents the apparent H_2 production yield normalized to the thermally oxidized plastic actually used in the ATT reaction (before mass correction). Y_{after} represents the overall H_2 production yield normalized to the initial plastic feedstock (after mass correction), reflecting H_2 production yield from the perspective of the original waste plastic input. Because $m_{ox} < m_{initial}$, Y_{before} is always greater than Y_{after}, and the two values diverge significantly when the extent of thermal oxidation mass loss is large. The relationship between the two is:

$$Y_{after} = Y_{before} \times (1 - \text{Mass loss (\%)} / 100) \quad \text{(Eq. S11)}$$

S1.4 Worked Numerical Example

The following example demonstrates the complete calculation procedure using representative experimental data. All values are taken directly from a single ATT experiment (oxidized PE at 300°C for 50 hours with a NaOH-to-plastic ratio of 5 in ATT).

Given values: F = 56.66 mL min^{-1}, t = 300 min, $\bar{C}_{H2}$ = 0.318 mol%, $\bar{C}_{N2,corr}$ = 99.68 mol% (air-corrected), $m_{initial}$ = 0.0588 g, m_{ox} = 0.05 g.

Step 3 (Eq. S5) — Mole fraction normalization:

$$\Sigma \bar{C}_{corr} = 0.318 + 99.68 = 99.998 \text{ mol\%}$$

$$x_{H2} = 0.318 / 99.998 = 0.003180, \quad x_{N2} = 99.68 / 99.998 = 0.99682$$

Step 4 (Eq. S6) — Moles of N_2 delivered:

$$n_{N2} = 56.66 \times 300 / 22.4 = 16{,}998 / 22.4 = 758.84 \text{ mmol}$$

Step 5 (Eq. S7) — Moles of H_2 produced:

$$n_{H2} = (0.003180 / 0.99682) \times 758.84 = 0.003190 \times 758.84 = 2.421 \text{ mmol}$$

Step 6 (Eq. S8–S11) — Mass loss and normalized H_2 yields:

$$\text{Mass loss (\%)} = (0.0588 - 0.05) / 0.0588 \times 100 = 0.0088 / 0.0588 \times 100 = 14.97 \text{ \%}$$

$$Y_{before} = 2.421 \text{ mmol} / 0.05 \text{ g} = 48.42 \text{ mmol g}^{-1} \quad \text{(Eq. S9)}$$

$$Y_{after} = 2.421 \text{ mmol} / 0.0588 \text{ g} = 41.17 \text{ mmol g}^{-1} \quad \text{(Eq. S10)}$$

Cross-check using Eq. S11: $Y_{after} = 48.42 \times (1 - 14.97/100) = 48.42 \times 0.8503 = 41.17$ mmol g^{-1}

Table S1. Optimized coordinates of product molecules (Figure S6) in xyz format.

20
PET-OH

C	-0.76165700	2.49774600	0.39958000
C	-2.86060500	1.42409900	-0.15099800
C	-0.07417800	1.29350200	0.21482000
C	1.45433600	1.16902400	0.42082700
C	-2.18040600	0.22018700	-0.33558600
H	-0.23692000	-0.75871400	-0.28801700
H	-2.66287300	3.50747800	0.36538900
C	-0.79730100	0.16020100	-0.15319300
H	-3.93657800	1.47544700	-0.29193100
H	-0.20032300	3.37823600	0.68830800
C	-2.14206800	2.56484700	0.21799200
H	-2.72910600	-0.67307500	-0.62296100
C	3.37423200	2.21270600	-0.61687200
O	1.97377500	2.23427500	-0.49387600
O	1.96806500	0.00768000	0.27022700
H	3.63984200	2.90160700	-1.42697500
H	3.87131400	2.54734400	0.30727800
H	3.73512500	1.20523000	-0.85823000
O	1.71847000	1.72474200	1.79232300
H	2.17764900	0.97612400	2.18912900

16
RCOOR-OH

C	-2.11675700	-0.60067700	-0.32291600
C	-1.09450000	0.36055800	0.33142600
H	1.65747800	0.74708200	0.78391300
O	0.14366500	0.08351400	-0.48475200
O	-1.41673900	1.59799400	0.42689000
H	-3.83820900	0.50121600	0.37097000
H	-4.22808400	-1.14811000	-0.17588200
H	-3.42356400	-0.88674300	1.38576200
H	1.96618700	0.81982600	-0.97157900
H	0.86323500	2.00361300	-0.20150000
C	1.20077000	0.95871300	-0.19767400
H	-2.19812600	-0.30182700	-1.37336000
H	-1.71888100	-1.62107500	-0.29765700
C	-3.48643800	-0.53421000	0.35179800
O	-0.77267200	-0.22978400	1.67763700
H	-0.86808400	0.55330600	2.23125500

15
RCOR-OH

C	-2.20371300	-0.69078500	-0.29058800
C	-1.09240800	0.29534900	0.20820700
C	0.23964000	-0.02182700	-0.53085900
O	-1.43352900	1.55885100	0.18040800
H	-3.80654600	0.62160700	0.28209300
H	-4.35460200	-1.04135100	-0.07030100
H	-3.50053200	-0.67452400	1.44516600
H	0.55532800	-1.06578200	-0.40417600
H	0.12435500	0.19160500	-1.60024100
C	-3.55395800	-0.43800100	0.37826100
H	-2.29871100	-0.54374400	-1.37534000
H	-1.88429000	-1.72913100	-0.12675800
H	1.01511500	0.63462600	-0.12704000
O	-0.81921400	-0.15951600	1.65660000
H	-0.95280600	0.68800100	2.09651600

13
RCOOH-OH

C	-2.09168700	-0.15276700	0.11781000
C	-1.10899800	1.01797300	0.13389600
H	-1.36309000	1.72792100	0.92970300
O	1.14476600	1.82517400	0.32653100
H	1.91249000	1.55219200	-0.18916500
H	-3.11420400	0.17801300	-0.10820000
H	-1.78301800	-0.87974100	-0.63900700
H	-2.09969500	-0.66049100	1.08657700
C	0.36068400	0.55837500	0.31300300
H	-1.15016300	1.56008700	-0.81663600
O	0.82778300	-0.29260000	-0.54299300
O	0.43844500	0.03968800	1.71113400
H	1.01692300	-0.71998800	1.57751800

12
RCHO-OH

C	-2.00749500	-0.18924600	0.11131800
C	-1.15502300	1.07818900	0.18646500
H	-1.46223500	1.70318800	1.03681100
H	0.88561700	1.78348400	0.35356000
O	0.86021100	-0.04215500	-0.59228900
H	-3.06604100	0.03578200	-0.07550400
H	-1.63262000	-0.82867800	-0.69267900
H	-1.93499400	-0.75226100	1.04668000
C	0.37286500	0.78211000	0.29411700
H	-1.29427800	1.67469300	-0.72623100
O	0.56447400	0.24756200	1.72118100
H	1.08105100	-0.53977100	1.51266300

Table S2. Ecoinvent database summary and environmental influences.

Name	Provider	CO_2 Emission Factor
CO_2 emission	Emission to air	1 kg-CO_2 eq./kg
NaOH	NaOH production | Cutoff, S - RoW	1.12 kg-CO_2 eq./kg
Na_2CO_3	Na_2CO_3 production | Cutoff, S - RoW	0.94 kg-CO_2 eq./kg
Electricity, high voltage	electricity production, hard coal | electricity, high voltage | Cutoff, S - RoW	0.47 kg-CO_2 eq./kWh
Heat, from steam, in chemical industry	steam production, as energy carrier, in chemical industry | heat, from steam, in chemical industry | Cutoff, S - RoW	0.18 kg-CO_2 eq./kg
Incineration (Direct CO_2 emission)	Stoichiometric calculation	Calculated
